# Positive Selection Pressure Drives Variation on the Surface-Exposed Variable Proteins of the Pathogenic *Neisseria*

**DOI:** 10.1371/journal.pone.0161348

**Published:** 2016-08-17

**Authors:** Jenny Wachter, Stuart Hill

**Affiliations:** Department of Biological Sciences, Northern Illinois University, DeKalb, IL, 60115, United States of America; National Cheng Kung University, TAIWAN

## Abstract

Pathogenic species of *Neisseria* utilize variable outer membrane proteins to facilitate infection and proliferation within the human host. However, the mechanisms behind the evolution of these variable alleles remain largely unknown due to analysis of previously limited datasets. In this study, we have expanded upon the previous analyses to substantially increase the number of analyzed sequences by including multiple diverse strains, from various geographic locations, to determine whether positive selective pressure is exerted on the evolution of these variable genes. Although *Neisseria* are naturally competent, this analysis indicates that only intrastrain horizontal gene transfer among the pathogenic *Neisseria* principally account for these genes exhibiting linkage equilibrium which drives the polymorphisms evidenced within these alleles. As the majority of polymorphisms occur across species, the divergence of these variable genes is dependent upon the species and is independent of geographical location, disease severity, or serogroup. Tests of neutrality were able to detect strong selection pressures acting upon both the *opa* and *pil* gene families, and were able to locate the majority of these sites within the exposed variable regions of the encoded proteins. Evidence of positive selection acting upon the hypervariable domains of Opa contradicts previous beliefs and provides evidence for selection of receptor binding. As the pathogenic *Neisseria* reside exclusively within the human host, the strong selection pressures acting upon both the *opa* and *pil* gene families provide support for host immune system pressure driving sequence polymorphisms within these variable genes.

## Introduction

The pathogenic *Neisseriae* possess two obligate human pathogens; *Neisseria gonorrhoeae* (Gc), the etiological agent of gonorrhea, and *Neisseria meningitidis* (Mc), which can cause bacterial meningitis [1]. For optimum infectivity, both neisserial pathogens need to form pili [2,3] as well as express Opa protein which serves as an afimbrial adhesion [4,5]. Both pathogenic species are capable of changing these surface antigens by one of two means; either by phase variation which causes on/off switching of gene expression, or via bona fide gene variation where the chemical composition of the surface antigen is modified [6]. In fact, the *opa* and *pil* genes of the pathogenic *Neisseria* spp. are perhaps the best studied prokaryotic systems of both phase and antigenic variation [6]. Both phase and antigenic variation can occur within the *pil* and *opa* systems, as multiple gene copies are maintained on the chromosome. Also, the *Neisseria* are naturally competent, allowing for the introduction of new alleles to a population. As these variable genes encode proteins that are surface exposed, each serves as an antigen that encounters immune surveillance. Consequently, it has been speculated that diversification of these multiple chromosomal genes is driven by selection pressures emplaced by the host immune system [7]. Due to this, a pathogen’s genes encoding antigenic proteins and the antigen-binding sites of immune genes are speculated to be under similar selection pressures, as each are in an evolutionary “arms race” for survival [8].

Type IV pili are primarily composed of PilE polypeptides encoded by the *pilE* expression locus [9]. The *pilE* gene varies through unidirectional recombination with one of several non-coding *pil* genes known as *pilS* that reside elsewhere on the chromosome. *Neisseria* harbor variable numbers of *pilS* loci, which may contain several *pil* gene copies at each locus (Gc strain MS11 contains 19 *pil* gene copies) [10–12]. Each *pilS* gene copy lacks the 5′ 150 bp of *pilE*, yet contains both constant and variable regions, with the variable regions (termed minicassettes; of which there are 6) being responsible for the formation of PilE antigenic variants following recombination of *pilE* with *pilS* [13]. Minicassette mc2, located towards the 3′ end of the gene, is regarded as the hypervariable segment of the *pil* genes, with this encoded segment of the PilE polypeptide experiencing the greatest exposure to the host immune system [14]. Consequently, the pilus filament, which is composed of thousands of copies of PilE polypeptide, is assembled in such a way that any conserved amino acid residues are shielded from the external environment [15].

In contrast to the *pil* system, the *opa* gene family consists of a variable number (11–12 in Gc and 3–4 in Mc) of unlinked, intact genes [5,16–21]. However, *opa* genes are not constitutively expressed at the protein level, because each *opa* gene contains a pentameric repeat segment within its coding region which allows for phase variation due to slipped-strand mispairing [16,22]. Opa proteins also contain three variable regions, denoted as hypervariable 1 (HV1), hypervariable 2 (HV2), and semivariable (SV) domains, which are surface-exposed as extracellular loops [19,23,24]. Opa proteins bind to human cell surface receptors which include CEACAM (CD66) and heparansulfate proteoglycans (HSPGs) [25–27]. It is believed that sequence changes within the SV and HV regions confer receptor specificities, with the CEACAM binding domain being determined by the HV1 and HV2 gene segments [28,29]. Facilitating Opa protein variability, *opa* genes exchange HV regions following horizontal transmission of DNA [30] which may result in unique Opa proteins occurring during an infection [20,31].

As both Opa proteins [32,33] and PilE polypeptide [34,35] constantly encounter the immune system, it has been assumed that variation of these gene sequences would be driven by strong immune selection pressure [8]. Thus, by maintaining nonsynonymous polymorphisms within the surface-exposed portions of antigen-encoding genes, and, by harboring multiple gene copies containing these distinct nonsynonymous polymorphisms, positive selection for such changes should allow these pathogens to better evade the host immune response. However, the presence of conserved regions within the genes, and the need to maintain protein structural integrity, may cause selection pressure to differ across the genes themselves. Additionally, Mc typically resides on the extracellular surface and must traverse the mucosal barrier of the nasopharynx in order to disseminate and colonize the meninges [36], while Gc characteristically inhabit the urogenital tract and rarely disseminate. Therefore, the difference between the extracellular and intracellular lifestyles of these pathogens may cause differential evolution of their extracellular proteins. In this study, the origins of polymorphisms in each system were investigated between species (i.e., between *N*. *gonorrhoeae* and *N*. *meningitidis*; termed interspecies), between multiple strains of a single species (either within *N*. *gonorrhoeae* or *N*. *meningitidis*; termed interstrain), as well as within a single strain of either *N*. *gonorrhoeae* or *N*. *meningitidis* (termed intrastrain). By differentially assessing the selective pressure on the variable genes from *Neisseria*, we demonstrate that immune pressure does indeed drive nonsynonymous changes within the variable gene sequences that encode the surface-exposed regions of the proteins, thus facilitating immune evasion.

## Materials and Methods

### Data acquisition

All sequences were obtained through the National Center for Biotechnology Information genome database (ftp://ftp.ncbi.nlm.nih.gov/genomes/Bacteria/) between April 14, and June 23, 2015. This analysis included *pil* and *opa* genes from 20 fully and partially sequenced strains of Gc along with 20 fully and partially sequenced strains of Mc (S1 Table). Annotated nucleotide and amino acid sequences obtained from these strains were used as a database to conduct a BLAST homology search (10^−3^ e-value cutoff) [37]. Collectively, the annotated and BLAST-inferred sequences included 381 *pil* genes and 309 *opa* genes from *Neisseria* (Gc: *pil n* = 237, *opa n* = 138; and, Mc: *pil n* = 144, *opa n* = 17).

### Amino acid and nucleotide alignments

Sequences were parsed based on characteristic elements of each gene (ie. the minicassettes of *pil*, the variable regions of *opa*). Such editing left 219 *pil* gene sequences (Mc: *n* = 71; Gc: *n* = 148), and 86 *opa* gene sequences (Mc: *n* = 17; Gc: *n* = 69). The strains and accession numbers of genes can be found in S1 Table and the sequences can be found in S1 and S2 Data. The parsed amino acid sequences were subjected to alignment utilizing MAFFT version 7.245 [38], allowing 2 alignments to be constructed between Pil and Opa sequences. These amino acid alignments were used to assemble nucleotide sequences into codon alignments through the use of PAL2NAL [39] (S1 Fig). For downstream analysis, phylogenies of the nucleotide alignments were constructed using FastTree version 2.1.8 which infers approximately-maximum-likelihood phylogenetic trees [40]. This program implements the general time reversible model (GTR) of nucleotide evolution [41] with the evolutionary rate heterogeneity determined through CAT approximation [42], and local support values calculated with the Shimodaira-Hasegawa test [43]. Although CAT likelihoods are typically higher than those calculated by the discrete gamma model, this method is considered an accurate estimator of evolutionary site rates for alignments >50 sequences. However, further inference utilizing this discrete gamma model, which rescales trees determined under the CAT approximation to optimize the Gamma likelihood under 20 different rate categories, revealed only slight differentiation among branch lengths between the CAT and gamma model trees. Additional construction of phylogenetic trees utilizing the GTR and HKY85 substitution models implemented in PhyML 3 [44] generated phylogenetic trees that were comparable amongst themselves, yet bore a slight variance in topology among trees constructed under Fasttree. As phylogenetic trees constructed under different methods in Fasttree and PhyML were comparable and only differed based on the program utilized in their construction, the datasets are presumed to be robust and differences in phylogenetic tree topologies are due to anomalies between the programs (S2 and S3 Figs). However, as FastTree is more accurate than PhyML 3 and other distance-matrix methods that are generally utilized for large datasets [40], trees generated by Fasttree were utilized for all downstream analysis.

### Calculation of polymorphisms and informative sites, d_*N*_ and d_*S*_

Synonymous and nonsynonymous DNA substitution rates were assessed using gene alignments subjected to polymorphism analysis by DnaSP version 5.10.1 [45] and the PAML version 4.8 suite of tools [46]. DnaSP analysis included the use of a sliding window approach to visualize both synonymous and nonsynonymous polymorphisms across both intra- and interspecies alignments which identified the number of singleton and parsimony informative sites for each alignment. The method of Yang and Nielsen, YN00 [47], was employed in the PAML suite to calculate the number of synonymous polymorphisms per synonymous site (*d*_S_) and the number of nonsynonymous polymorphisms per nonsynonymous site (*d*_N_). This method is more reliable than those proposed by Nei and Gojobori [48] and Ina [49] as, unlike other approaches, both the HKY85 [50] and F84 [51] models are utilized to account for the transition/transversion rate bias and codon frequency when calculating the *d*_S_ and *d*_N_ through an approximation method. Additionally, baseml, another program within the PAML suite, was utilized to determine the rate of nucleotide substitutions [47]. Baseml analysis involved the application of seven different nucleotide substitution models (JC69, K80, F81, F84, T92, TN93, and REV) toward the *pil* nucleotide alignment in order to determine the best-fit model for the dataset by determining the 2δ of the upper limit of the log-likelihood from each derived tree.

### Estimation of substitution saturation

As substitution saturation of one or more sequences causes a loss of phylogenetic information that can lead to erroneous conclusions, the test of substitution saturation implemented by the DAMBE program was applied toward both the *opa* and *pil* nucleotide alignments [52–54]. Substitution saturation occurs when nucleotide substitutions within a site occur so frequently that the position is said to be saturated with polymorphisms, when this occurs, a true phylogenetic tree can no longer be assembled from the dataset. This test of substitution saturation calculates a critical index of substitution (I_SS.C_) which serves as the cut-off value in which sequences will no longer produce the true phylogenetic tree. The I_SS.C_ value is calculated from the critical tree length (the tree length with a probability ≥ 0.95), the sequence length of the alignment, and the number of operational taxonomic units. The I_SS.C_ can be compared to the index of substitution saturation (I_SS_) calculated from the data, with a significantly smaller observed I_SS_ value, as determined by a two-tailed Student’s T test, indicating that the data is not affected by substitution saturation and is suitable for phylogenetic analysis. As the sensitivity of the method in detecting phylogenetic signals is reduced by unresolved sites and those containing gaps, only fully resolved *opa* and *pil* sites were used for the calculation of the I_SS._ To determine the operational taxonomic units (OTUs) within the *opa* and *pil* datasets, the 16S rRNA sequences from each *Neisseria* strain were aligned with MAFFT [38], and sequences that contained < 97% identity were considered as a separate OTU. This analysis identified a total of three different OTUs within the *Neisseria* dataset (data not shown).

### Detection of recombination

To determine whether the detected polymorphisms arose due to frequent recombination, the standard measure of linkage disequilibrium, Dʹ, was estimated for each alignment [55]. The ratio of significant phylogenetically informative sites (*p* < 0.05) to the number of possible pairwise comparisons was used to determine if the number of detected sites would be expected under pure randomness. This test was performed with DnaSP [45]. Putative gene conversion events between *pil* and *opa* were detected using Geneconv v. 1.81 [56,57] and displayed with Circos [58]. Geneconv determines the length of fragments that contain similar quantities and patterns of polymorphisms between paired alleles. Polymorphic sites of paired alleles are then randomized 10,000 times to generate a random sample of fragments given the observed number of polymorphic sites. The observed fragments are then compared with the permuted lengths to determine whether they are longer than expected by chance and to compute *p* values. Statistically significant gene conversion fragments were further parsed to remove any detected recombination events that occurred entirely within constant regions.

### Computational analysis for the determination of sites under selective pressures

To determine sequence divergence from neutral expectations, mathematical tests that apply the neutral theory of evolution as the null hypothesis were employed. These “tests of neutrality” can provide insights into intraspecific (Tajima’s D) and interspecific (*d*_N_/*d*_S,_ Poisson Random Field Model, phylogenetic analysis by maximum likelihood) selection pressures [59–61]. Tajima’s test, the Poisson Random Field Model (PRFMLE) and phylogenetic analysis by maximum likelihood (PAML) were used to estimate the direction of selection pressures acting on the *pil* and *opa* genes.

The Tajima test uses the *D*_T_ statistic, which is computed from the difference in the expectation (*S*) and the variance of the average number ($\hat{k}$) of (pairwise) nucleotide differences between DNA sequences [62]. By applying the Tajima test via DnaSP, a sliding window approach allowed calculation of *D*_T_ along various intervals in the alignments [45]. Additionally, the M7 and M8 site models of CODEML (in the PAML suite) were also employed [63–65]. All site models use the codon rather than a single nucleotide as the unit of evolution [63]. The M7 model cannot account for positively selected sites (*d*_N_/*d*_S_ ≤ 1), while the alternative M8 model allows for positive selection (*d*_N_/*d*_S_ > 1)_._ Positive selection is indicated if the M8 likelihood score is significantly better than that of M7. Likelihood ratio tests (LRTs) were performed on the log-likelihoods in order to compare the levels of significance between the null (M7) and alternative (M8) models [64]. The Poisson Random Field Model (PRF) [66], applied through the PRFMLE program, estimates the intensity of selection on synonymous and nonsynonymous sites [60,67]. The PRF model estimates the expected number of polymorphic sites in a population [66,68,69], permitting the values of the per locus mutation rate parameter (μ) and the scaled selection pressure (γ) to be estimated through a likelihood equation. The unscaled value of γ would be equal to the selective advantage of the cell expressing a given gene with the sign (+ or -) of the variable conveying the direction of selection pressure [7].

### Structural prediction of conserved and variable regions

Protein structures of Pil and Opa were obtained from RCSB Protein Data Bank (PDB) [70]. These structures, along with the constructed protein alignments and phylogenetic trees, were used to visualize regions determined to be under positive selection with The Consurf Server [71–74]. The structures of Pil 2HIL [15] and Opa 2MAF [75] were used in this analysis. Protein structures were depicted with UCSF Chimera v 1.10.2 [76].

### Calculation of solvent accessible surface areas

In order to determine solvent exposed regions of strain MS11 Opa (2MAF) [75] and strain C30 (a variant of strain MS11) Pil (2HIL) [15] proteins, GETAREA, a subroutine of the FANTOM program was utilized [77]. This program efficiently calculates the solvent accessible surface area through analysis of Cartesian coordinates stored in PDB format files. Additionally, the continuum solvation models implemented by this program were determined to produce protein conformations containing lower total energy values than those calculated from the empirically derived parameters of other methods [78,79].

## Results

### Intra- and interspecies homology varies among *Neisseria* genes

**Fig 1:**
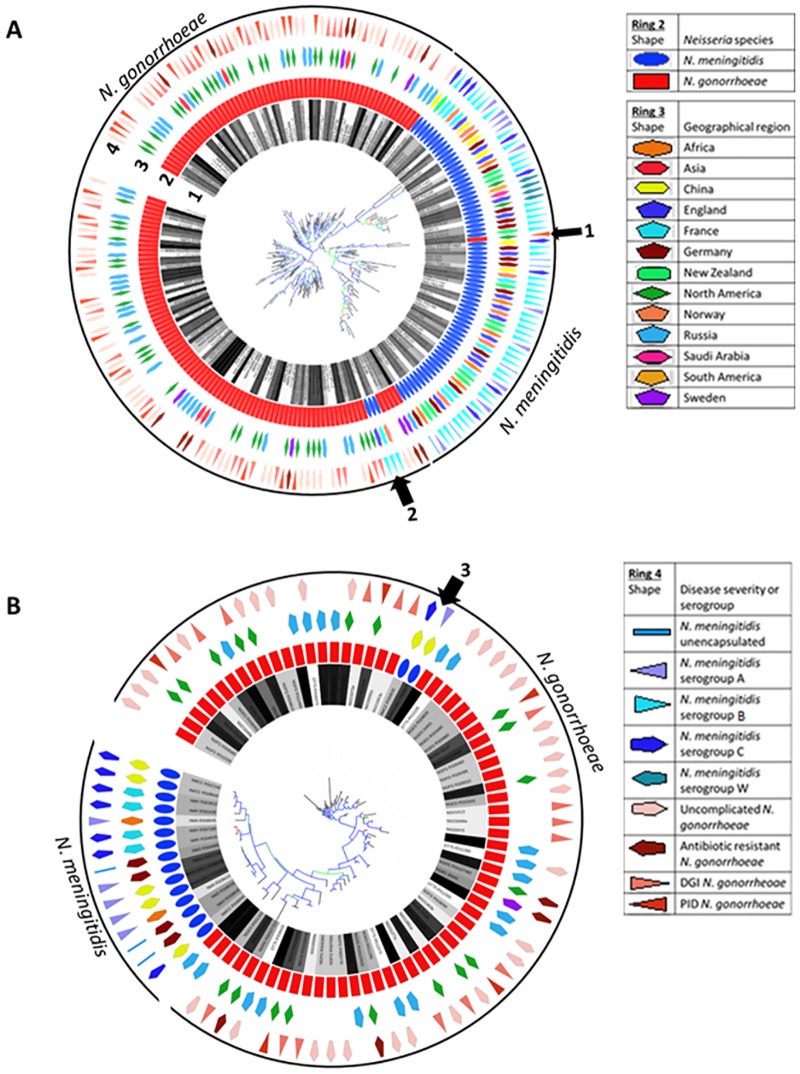
*pil* and *opa p*hylogenetic trees. (a) 219 *pil* genes analyzed; (b) 86 *opa* genes analyzed. The trees depicted were determined using the GTR model of Fasttree. The colored boxes closest to the trees indicate the strain of *N*. *gonorrhoeae* or *N*. *meningitidis* (ring 1), while the second ring signifies the species of the isolate, either *N*. *gonorrhoeae* or *N*. *meningitidis*. The third ring denotes the geographical region of the isolate, when known, while the outermost ring indicates the severity of the disease or the serogroup of the strain. The bootstrap values as calculated with the Shimodaira-Hasegawa test are depicted as colored branches, where red branches indicate minimum bootstrap values (0), green indicate median bootstrap values, and blue indicate maximum bootstrap values (1). While the polymorphisms, insertions, and deletions within the majority of *pil* and *opa* genes are unique to a single species (either *N*. *gonorrhoeae* or *N*. *meningitidis*; as indicated by the solid lines above the figures), there is a single *pil* gene from Gc indicated by arrow 1 and three from Mc indicated by arrow 2 in (a) and two *opa* genes from Mc indicated by arrow and 3 in (b) that display more homology to genes from the opposite species.

Phylogenetic trees constructed from nucleotide sequence alignments can provide insights into gene divergence based upon sequence polymorphisms, insertions and deletions. Therefore, *pil* and *opa* phylogenies reveal a high degree of divergence between the two *Neisseria* species with both genes forming a species-specific cluster (Fig 1A and 1B). However, within a species, sequence conservation between isolates from similar geographic locations, disease manifestations (Gc), or serogroups (Mc) is relatively weak. Instead, strains of Gc and Mc from distinct locales share few homologous genes (third ring in Fig 1), implying that the observed polymorphisms within *pil* and *opa* have occurred independently, following the divergence of the two species, yet not before allowing polymorphisms to emerge prior to strain differentiation (colored boxes closest to the tree in Fig 1) and geographical isolation (third ring in Fig 1). The majority of *pil* and *opa* genes exhibit intraspecific homology, with a single *pil* gene of Gc and two *pil* genes of Mc (arrow 1; Fig 1A), along with two *opa* genes of Mc (arrow 3 in Fig 1B) serving as outliers.

### Lack of substitution saturation validates phylogenetic analysis of *opa* and *pil*

Despite the high degree of polymorphisms identified within the variable *opa* and *pil* genes, analysis indicated that the index of substitution saturation calculated for both was significantly less (*p* < 0.0001) than the critical index of substitution saturation (S2 Table; corresponding to the closest number of OTUs determined for the analyzed pathogenic *Neisseria* strains, OTU = 4). Therefore, neither *opa* nor *pil* have experienced an excess of sequence polymorphisms required to drive sequences past the critical index of substitution saturation. This indicates that any conclusions drawn from the alignments and phylogenetic analyses are not rendered invalid due to substitutions present within these sequences.

### A positive selection-dominated landscape in *Neisseria* variable genes

**Fig 2:**
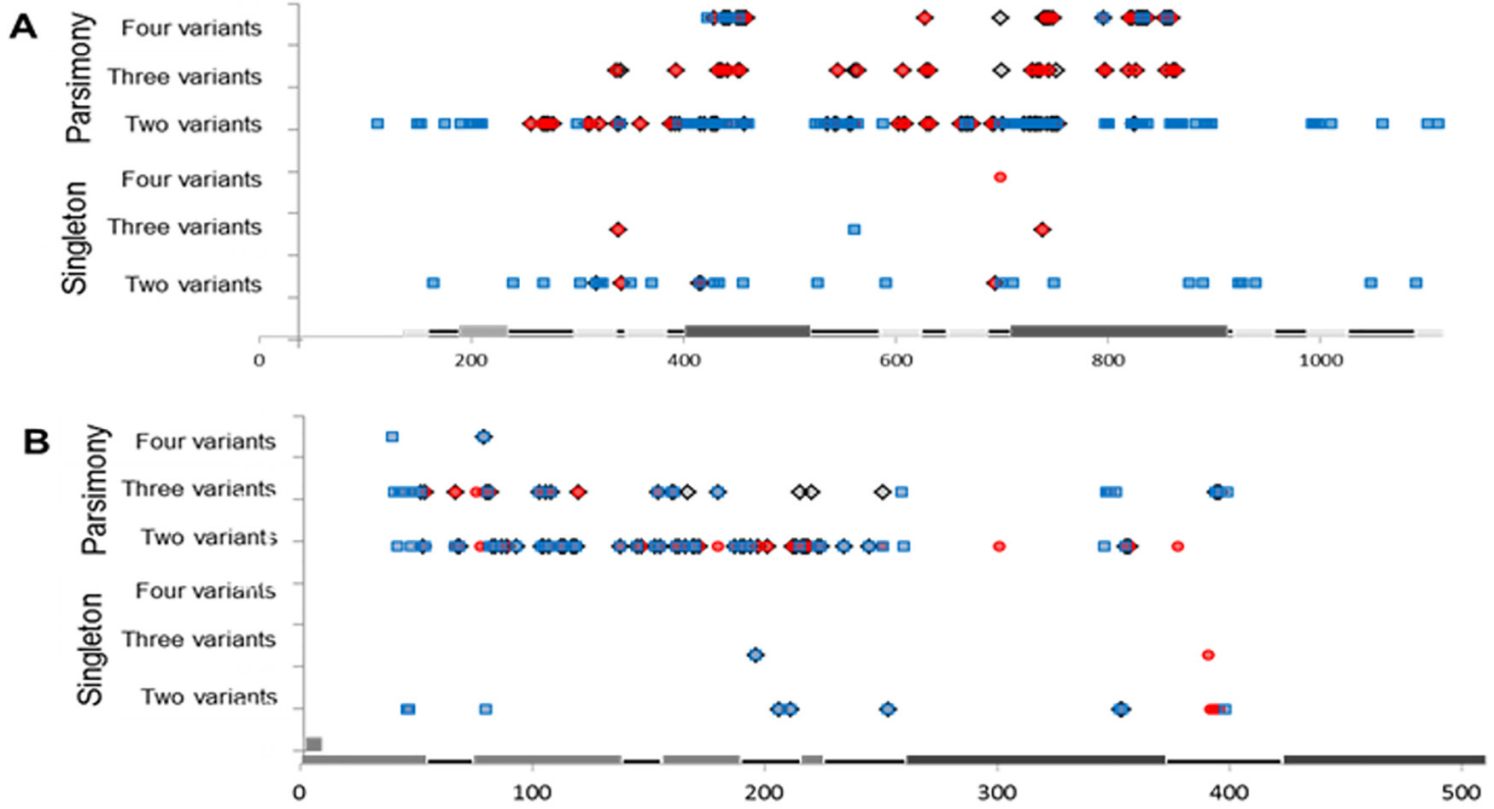
Location of polymorphisms within nucleotide alignments. Nucleotide alignments of (a) 69 GC *opa* genes and 17 MC *opa* genes and (b) 148 GC *pil* genes and 71 MC *pil* genes. Red circles indicate intra-species polymorphisms within Gc (a and b), blue squares indicate intra-species polymorphisms within Mc (a and b), and black diamonds indicate inter-species polymorphisms present within *opa* and *pil* genes of Gc and Mc (a and b). The lines along the bottom indicate the conserved regions (black line), the variable regions (dark gray boxes), and for *opa*, the membrane-spanning regions (light gray boxes).

As *pil* [10,13] and *opa* [24,80] sequences are known to contain conserved and variable regions, protein alignments were constructed in order to delineate the conserved segments. Sliding window analysis revealed that the majority of detected polymorphisms occurred within two or more gene sequences and were therefore parsimony-informative (Fig 2). These parsimony-informative polymorphisms were detected throughout the length of the *opa* (Fig 2A) and *pil* (Fig 2B) alignments. Consequently, nucleotide sequence variation is not restricted to just the variable regions of the genes.

The array of parsimony-informative sites within the *opa* and *pil* genes were then used to differentiate between synonymous and nonsynonymous polymorphisms. Determining the number of sequences that carry a synonymous or nonsynonymous polymorphism allows for the *d*_N_ and *d*_S_ variables for that sequence alignment to be calculated, thereby permitting the estimator of selection pressure, the *d*_N_/*d*_S_ ratio, to be ascertained. As nonsynonymous polymorphisms result in a change to the resulting amino acid sequence, these polymorphisms are assumed to occur under selective conditions. Consequently, a *d*_N_/*d*_S_ ratio greater than one is an indicator of positive selection. Examination of *d*_N_ and *d*_S_ in the *opa* and *pil* alignments revealed that there was a greater number of nonsynonymous than synonymous changes present in interspecies polymorphisms of *pil* and *opa* (~1.27 for *pil* and ~1.79 for *opa*; Table 1). Therefore, positive selection appears to have influenced the divergence of these genes.

**Table 1:** Intra- and interspecies analysis of polymorphisms present within the *pil* and *opa* genes of Mc and Gc.

Gene		*d*_N_	*d*_S_	*d*_N_/*d*_S_	Type of alignment analyzed (interspecies or interstrain)
*pil n* = 219		0.0748	0.0589	1.2708	Interspecies
	*N*. *meningitidis n* = 71	0.1044	0.0984	1.0613	Interstrain
	*N*. *gonorrhoeae n* = 148	0.1037	0.0229	4.5236	Interstrain
*opa n* = 86		0.2602	0.1452	1.7916	Interspecies
	*N*. *meningitidis n* = 17	0.1300	0.1092	1.1896	Interstrain
	*N*. *gonorrhoeae n* = 69	0.3842	0.5355	0.7174	Interstrain

### Differential biases in synonymous and nonsynonymous polymorphism locations

**Fig 3:**
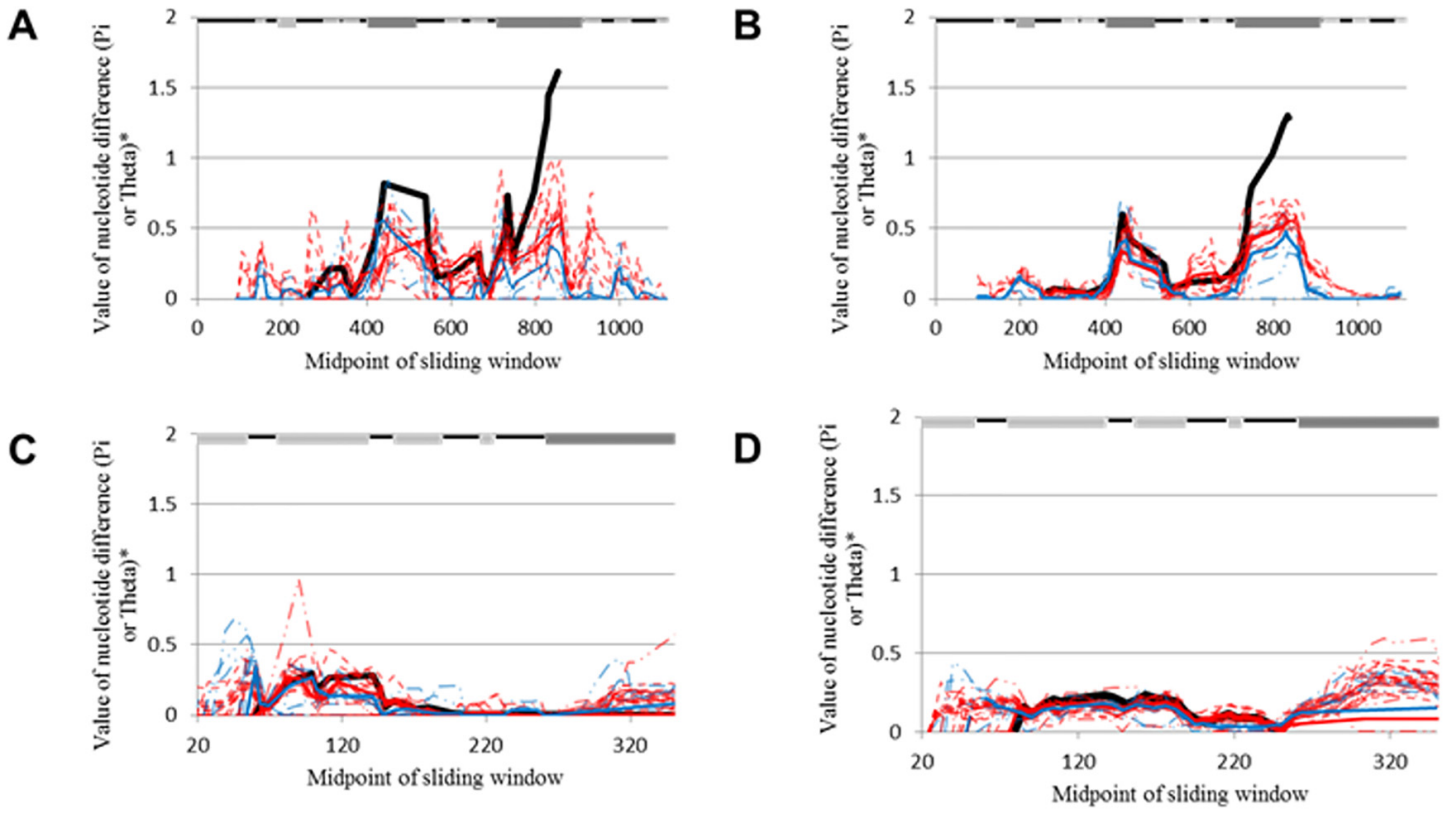
Location of synonymous and nonsynonymous polymorphisms. Nucleotide alignments of *opa* (Gc = 69 and Mc = 17) and *pil* (Gc = 148 and Mc = 71) were analyzed for the presence of synonymous (*opa*, panel a and *pil*, panel c) and nonsynonymous polymorphisms (*opa*, panel b and *pil*, panel d). The graph demonstrates the diversity values (Pi or Theta) present amongst sequences based on the location in the nucleotide alignment. The nucleotide diversity occurring between Gc and Mc (thick black line), within Gc (red line), within Mc (blue line), and within each species of Gc (dashed red lines) and Mc (dashed blue lines) are shown. The lines along the top indicate the conserved regions (black line), the variable regions (dark gray boxes), and the *opa* membrane-spanning regions (light gray boxes). A larger number of both synonymous and nonsynonymous polymorphisms can be seen within the variable regions of *opa* within Gc and Mc. Compared to *opa*, relatively few polymorphisms are present within *pil*, however, the polymorphisms present occur within the variable mini-cassettes.

**Fig 4:**
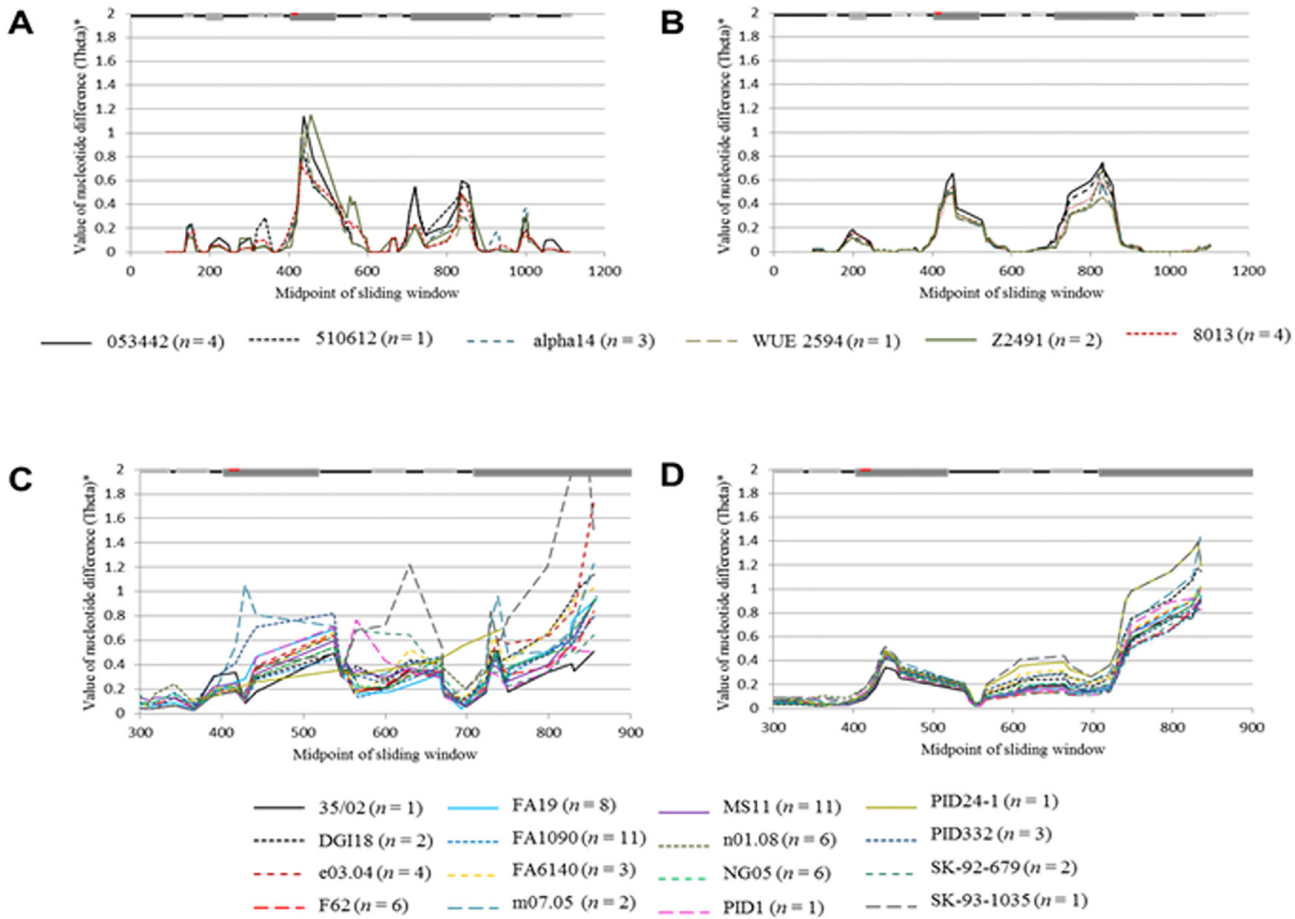
Location of interstrain synonymous and nonsynonymous polymorphisms within nucleotide alignments of *opa*. Nucleotide alignments of *opa* from Mc (*n* = 17) and Gc (*n* = 69) were analyzed for synonymous (Mc, panel a, Gc, panel c) and nonsynonymous (Mc, panel b, Gc, panel d) polymorphisms. The graph demonstrates the diversity values (Theta) present amongst sequences based on the location in the nucleotide alignment. The nucleotide diversity occurring between species is demonstrated below the figures. The lines along the top indicate the conserved regions (black line), the variable regions (dark gray boxes), and the membrane-spanning regions (light gray boxes). While the synonymous polymorphisms occurring within *opa* appear to be dependent on the strain of Mc or Gc, nonsynonymous polymorphisms occur within the same regions of the nucleotide alignment, regardless of species, and are almost exclusive to the variable regions of *opa*.

**Fig 5:**
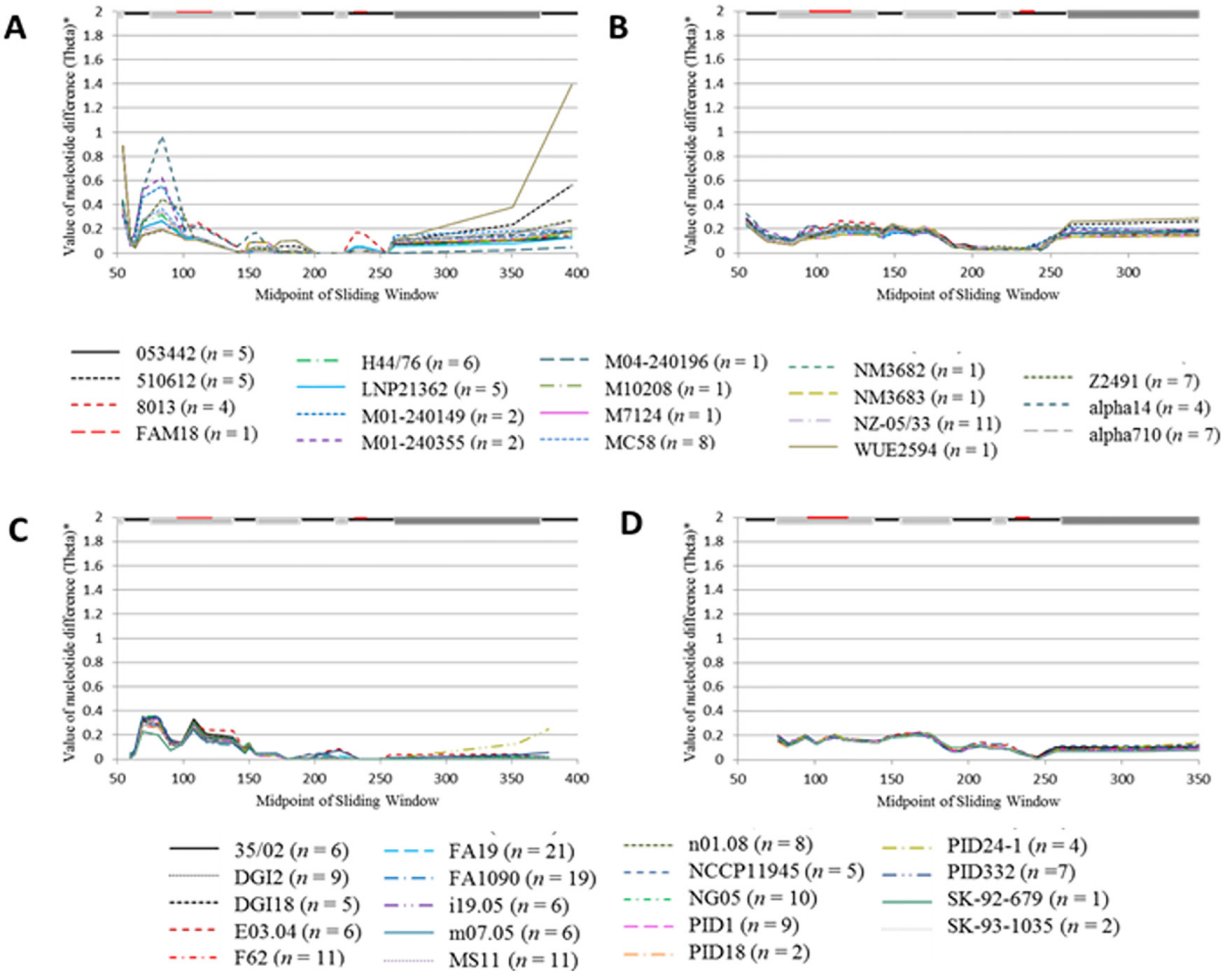
Location of interstrain synonymous and nonsynonymous polymorphisms within nucleotide alignments of pil. Nucleotide alignments of *pil* from Mc (*n* = 71) and Gc (*n* = 148) were analyzed for synonymous (Mc, panel a, Gc, panel c) and nonsynonymous polymorphisms (Mc, panel b, Gc, panel d). The graph demonstrates the diversity values (Theta) present amongst sequences based on the location in the nucleotide alignment. The nucleotide diversity occurring between species is demonstrated below the figures. The lines along the top indicate the conserved regions (black line), and the variable regions (dark gray boxes). The *pil* genes of Mc display slightly different synonymous polymorphisms near the 5′ end of the nucleotide alignment, however, the majority of *pil* genes within Mc and Gc possess similar locations containing synonymous and nonsynonymous polymorphisms.

A sliding window approach was used to graphically depict the quantity of synonymous and nonsynonymous polymorphisms within the analyzed genes. The majority of polymorphisms (either synonymous or nonsynonymous) lie within the variable regions of *opa* (Fig 3A and 3B). However, compared to *opa*, relatively few polymorphisms are present within *pil* (as indicated by the height of the peaks in Fig 3C and 3D), yet, when present, they also occur within the variable regions, as seen in the minicassettes. Examination of interstrain polymorphisms revealed that while the amount and location of synonymous polymorphisms differ amongst species (as indicated by the strain-specific lines in Figs 4A, 4C, 5A and 5C), the extent and position of nonsynonymous polymorphisms within *opa* (Fig 4B and 4D) and *pil* (Fig 5B and 5D) are fairly homologous (as seen by the similar location and intensity of polymorphisms of strain-specific lines).

Further analysis of interstrain polymorphisms revealed that the nonsynonymous polymorphisms detected within Mc *opa* genes occurred almost exclusively within the three variable regions of the gene, with the majority occurring within HV1 and HV2 (Fig 4B). In contrast, Gc *opa* genes lacked any detected sequence polymorphisms at the 5′ end of the alignment, even within the semivariable region (due to this Fig 4C and 4D begin downstream of the semivariable region at nucleotide 300 in the alignment, which allows greater expansion of the sliding window on the x-axis). Despite this, the nonsynonymous polymorphisms present within each Gc strain followed a similar pattern, with the majority of polymorphisms occurring within the two hypervariable domains. However, some nonsynonymous polymorphisms were observed within the membrane spanning region between the two HV regions. Overall, this analysis demonstrates almost exclusive selection for protein-altering substitutions occurring within the exposed hypervariable domains in both Mc and Gc, implying selection pressures within these regions (as evidenced in the *d*_N_/*d*_S_ ratio of ~1.79, Table 1).

Within *pil* genes, both Gc and Mc contain higher levels of synonymous substitutions near the 5′ end of the variable regions (within mc5; Fig 5A and 5C) and display slightly elevated levels of nonsynonymous substitutions throughout the variable regions (Fig 5B and 5D). Therefore, the majority of base pair substitutions detected within the polymorphism analysis (Fig 2b) are synonymous within the 5′ end of *pil*. The comparative absence of nonsynonymous substitutions within the *pil* variable regions may be due to the high degree of heterogeneity, which may cause non-continuous alignments for analysis. Nonetheless, the number of detected nonsynonymous polymorphisms within *pil* is still sufficient to skew the *d*_N_/*d*_S_ ratio in favor of positive selection as the calculated *d*_N_/*d*_S_ ratio is ~1.27 (Table 1).

### Analysis of linkage equilibrium within the variable genes of *Neisseria* spp.

Evidence exists for horizontal gene transfer occurring within *opa* [20] and intragenic recombination with *pil* [81,82]. Consequently, the standard measure of linkage equilibrium, D′, can be estimated. Alleles are said to be under linkage equilibrium if D′ is less than the 0.05 proportion that would be expected under pure randomness. Estimation of D′ for *opa* and *pil* using the two-tailed Fisher exact test revealed that frequent recombination does not account for the detected polymorphisms occurring among species, as both interspecies and interstrain analysis show evidence for linkage disequilibrium (Table 2).

**Table 2:** Linkage disequilibrium detected within the interspecies alignment of *pil* and *opa* genes.

Gene		Region analyzed	Possible pairwise comparisons	Statistically significant pairwise comparisons (P < 0.05)	D′	Type of alignment analyzed (intra- or interspecies)
*pil* (*n* = 219)		Entire alignment	1378	238	0.17	Interspecies
		mc1	No pairwise comparisons			Interspecies
		mc2	10	2	0.20	Interspecies
		mc3	10	7	0.70	Interspecies
		mc4	28	12	0.43	Interspecies
		mc5	120	40	0.33	Interspecies
		mc6	No pairwise comparisons			Interspecies
	*N*. *meningitidis n* = 71	Entire alignment	1225	158	0.13	Interstrain
	*N*. *gonorrhoeae n* = 148	Entire alignment	1326	252	0.19	Interstrain
*opa* (*n* = 86)		Entire alignment	2485	521	0.21	Interspecies
		SV	No pairwise comparisons			Interspecies
		HV1	45	10	0.22	Interspecies
		HV2	190	81	0.43	Interspecies
	*N*. *meningitidis n* = 17	Entire alignment	8385	1159	0.14	Interstrain
	*N*. *gonorrhoeae n* = 69	Entire alignment	2556	503	0.20	Interstrain

The alignments of the entire length of *pil* (mc6-mc1) and *opa* as well as the individual *pil* minicassettes (mc1-6) and *opa* variable regions (SV and HV1-2) were analyzed for evidence of linkage disequilibrium. The number of possible pairwise comparisons and the number of statistically significant pairwise comparisons as determined by the two-tailed Fisher exact test were used to determine D′.

The presence of constant regions within the gene sequences may have skewed the above analysis. Consequently, the variable regions were analyzed separately to determine if polymorphisms encountered within exposed regions are attributable to recombination. However, such independent analysis of the variable regions did not differ from the previous inference as *opa* SV, HV1-2, and *pil* mc1-6 were found to be under linkage disequilibrium among and within species (Table 2). However, significant D′ values were detected between the majority of intrastrain *opa* (~88% of strains analyzed) and *pil* (~97% of strains analyzed) genes analyzed. This indicates that the majority of Gc and Mc strains contain *opa* and *pil* genes that are in linkage equilibrium (S3 and S4 Tables).

**Fig 6:**
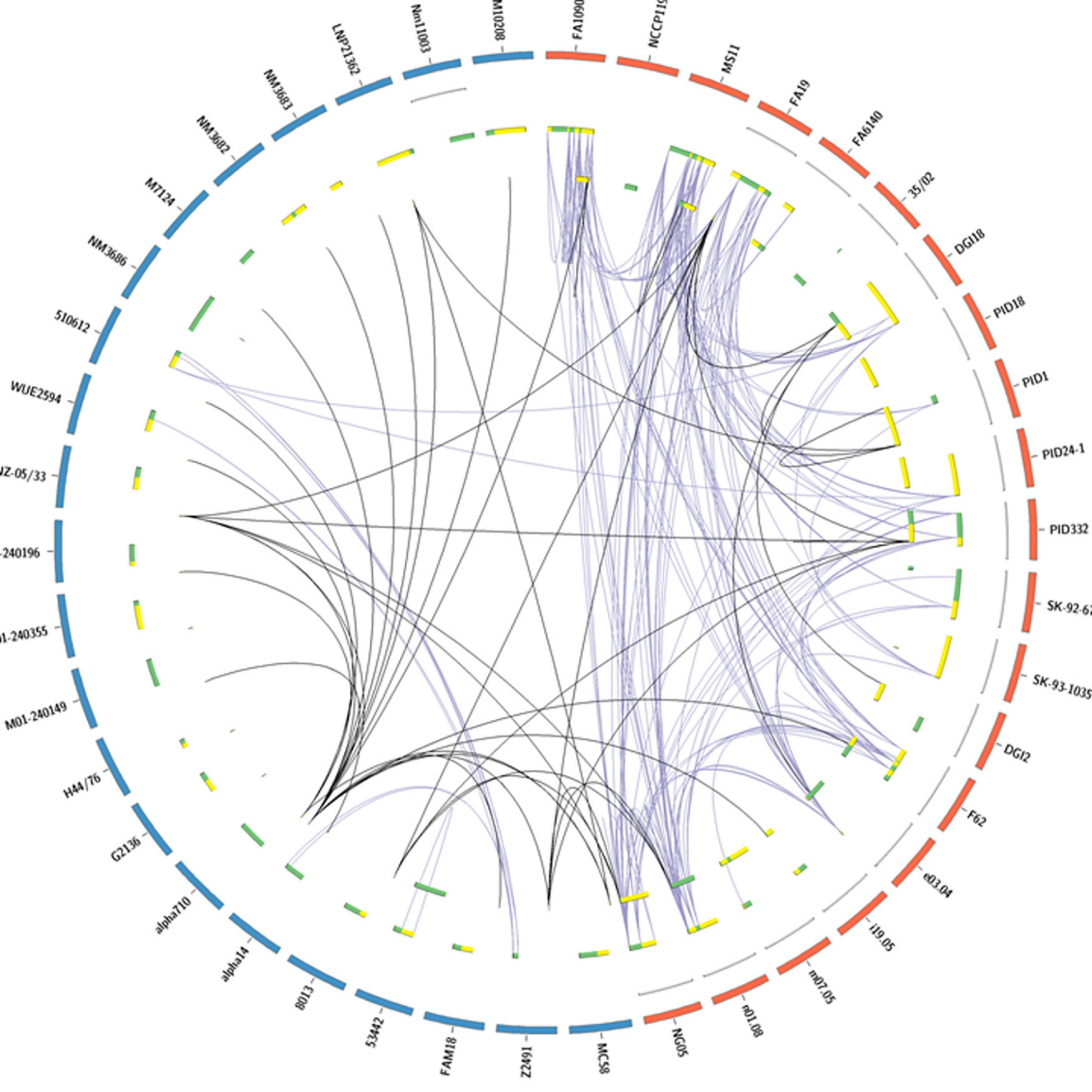
Linkage map of detected *pil* and *opa* recombination events. Geneconv was used to detect statistically significant evidence (*p* < 0.05) for gene conversion events between the *pil* and *opa* genes of Gc and Mc [56]. Statistically significant gene conversion fragments were further parsed to remove any implied recombination events that occurred entirely within constant regions of the genes. Chromosomes of Mc are shown in blue while chromosomes of Gc are shown in red, all chromosomes are denoted by the strains of Mc or Gc they represent. Any scaffold chromosomes (assembled as contigs) are shown as thin grey lines inset to the chromosomes they represent. *opa* are shown as either green (sense) or yellow (antisense) bars relative to their locations on the chromosome. *pil* are shown inset to *opa* as either green (sense) or yellow (antisense) bars relative to their locations on the chromosome. Statistically significant gene conversion events are displayed as lines connecting either *opa* (purple) or *pil* (black) genes.

In order to detect any interspecies, as well as inter- and intrastrain horizontal gene transfer events between these variable genes, the Sawyer test was applied using Geneconv [56,57]. This analysis revealed evidence for recombination between the variable regions of both *pil* and *opa* (Fig 6), with the majority of these recombination events occurring between strains of *Neisseria* (interspecies = ~8%, interstrain = ~83%, and intrastrain = ~9%). Despite the fact that *opa* contained more inter- and intrastrain recombination events (~81% of all interstrain and ~76% of all intrastrain recombination events), *pil* presented more detectable interspecies recombination events (~89% of all interspecies recombination events that were detected). Furthermore, while the majority of these interspecies events were due to recombination of variable regions (~97% in *opa* and 100% in *pil*), a small portion of allelic exchanges were detected within *opa* (~3%). Therefore, these data indicate that *pil* is exchanged more often between species while *opa* transfer is more prevalent between and within strains. However, due to the fact that all interspecies and interstrain *opa* and *pil* genes are in linkage disequilibrium (Table 2), the majority of these detected recombination events (~91%) do not account for the polymorphisms detected in this analysis.

### Confirmation of selection pressures within the variable genes of *Neisseria*

**Fig 7:**
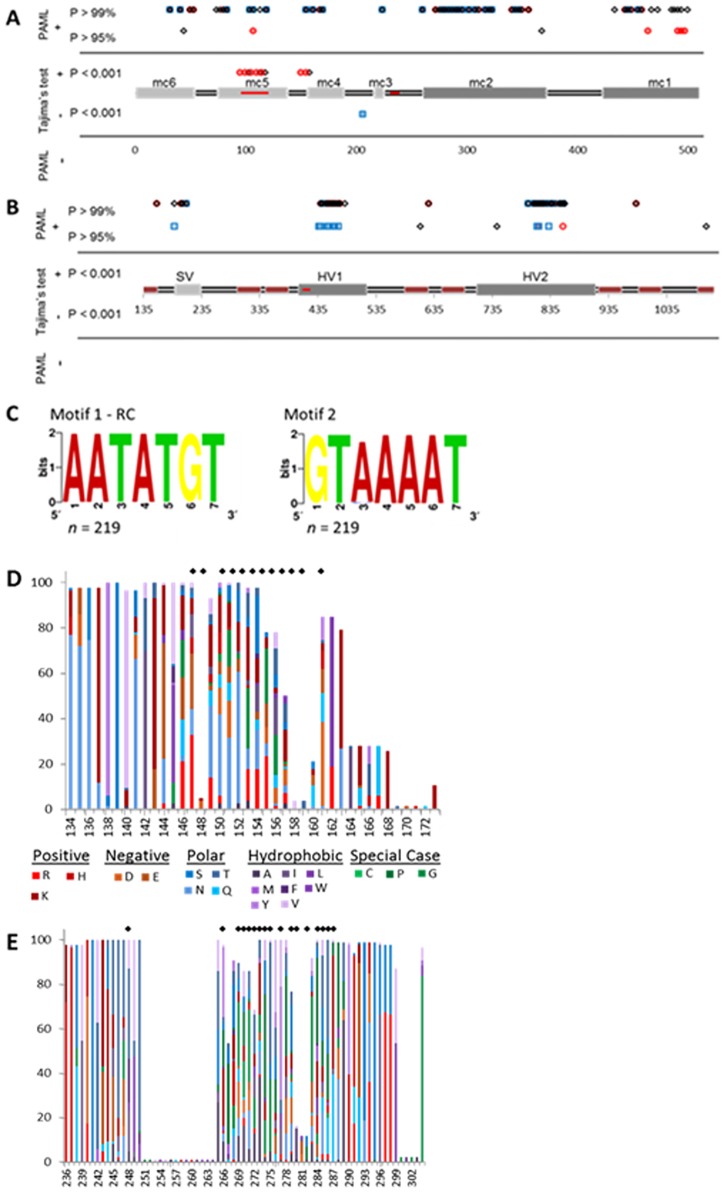
Location and analysis of detected selection pressures within *opa* and *pil*. Alignments of *opa* (a) and *pil* (b) from Gc (*opa*, *n* = 69; *pil*, *n* = 148) and Mc (*opa*, *n* = 17; *pil*, *n* = 71) were analyzed for selection pressures using the Bayes Empirical Bayes analysis implemented under the alternative model of PAML and Tajima’s test. The figure demonstrates the statistically relevant locations exhibiting either positive or negative selection pressures detected under each test for interspecies (black diamonds) and interstrain alignments of Gc (red circles) and Mc (blue squares). The lines along the bottom indicate the conserved regions (black line), and the variable regions (gray boxes), and for *opa* the membrane spanning regions (red boxes). The majority of positively selected sites within *pil* and *opa* occur within the variable regions. Two *pil* non-canonical promoter motifs detected from small transcriptome studies of *N*. *gonorrhoeae* strain MS11 were found to be highly conserved among the 219 *pil* genes analyzed, as shown by sequence logos (c) in which the height of the nucleotides in the logo demonstrate their conservation among *pil*. Their locations within *pil* are shown by black bars in mc5 and the constant region between mc3 and mc2 (b). The relative percentage of amino acids present within the HV1 (d) and HV2 (e) variable regions of the Opacity proteins. The positively selected regions within HV1 and HV2 are shown as diamonds above their respective amino acid positions. As can be seen, nonsynonymous polymorphisms are highly prevalent within these positively selected regions with the amino acids present within these regions being highly varied.

Various tests were employed to determine if aligned sequences fell under the neutral mutation hypothesis. Tajima’s test was used to determine both inter- and intrastrain selection pressures. Analysis of entire alignments did not reveal significant evidence for selection, save for purifying selection within the *opa* genes of Gc strain e03.04 (data not shown). However, sliding window analysis revealed statistically significant (*p* < 0.05) positive selection within the interstrain analysis of Gc *pil* (71% within minicassettes), while purifying selection was detected within the interstrain analysis of Mc *pil* (100% within constant regions) (Fig 7B and Table 3). All other statistically significant *D*_T_ values (*p* < 0.05) denoted purifying selection of intrastrain *opa* (Mc = 1, Gc = 4) and *pil* (Mc = 5, Gc = 4). Of the sites predicted to be under the influence of purifying selection, less than half (*opa* = 18%, *pil* = 24%) lie within the predefined constant regions indicating a functional role for these sites (Table 3). In fact, previous data has identified the presence of *pil*-derived small RNAs (sRNAs) arising from experimentally verified non-canonical promoter elements in Gc strain MS11 *pilS*6 and *pilE* loci [83–85]. Further investigation into the Gc MS11 small transcriptome identified two non-canonical promoter motifs upstream of other *pil*-derived sRNAs (data not shown). Examination among the *pil* genes of pathogenic *Neisseria* revealed strong conservation of these promoter motifs (Fig 7C) as homologs were identified within every *pil* gene examined (*n* = 219). However, investigation into selection pressures utilizing non-coding nucleotide substitution analysis revealed heterogeneity (α = 0.12) among the entire *pil* alignment utilizing the general-time-reversible model (REV) as the best-fit model to our dataset (as calculated by the 2δ of the log likelihoods; data not shown). Therefore, the relative heterogeneity of *pil* within the locations harboring the motifs was analyzed independently. While motif 1 lies within a variable minicassette (mc5; shown by a black bar in Fig 7B), motif 2 is contained within a conserved region of the gene and, therefore, putative promoters within this region were found to be highly conserved (~97%; Fig 7C and indicated by a black bar between mc3 and mc2 in Fig 7B). Furthermore, although codon substitution models were utilized, about 16% of variable sites determined to be under purifying selection contain promoter motif 1 (as indicated by arrow 1 in Fig 7B) all of which contain a perfectly conserved non-canonical promoter sequence (5′-AATATGT-3′) indicating that this portion of the variable minicassette is most likely placed under purifying selection at the nucleotide level in order to maintain this promoter element [85].

**Table 3:** Portions of Mc and Gc *opa* and *pil* genes determined to be under the influence of selection with Tajima’s test.

Gene	Inter- or Intrastrain analysis	Species/strain	Selection	Number of detected sites	% of detected selection within constant regions
*opa*					
	Intrastrain	Mc 053442 *n* = 4	Purifying	16	13
	Intrastrain	Gc FA1090 *n* = 11	Purifying	1	100
	Intrastrain	Gc FA19 *n* = 8	Purifying	1	100
	Intrastrain	Gc MS11 *n* = 11	Purifying	4	100
	Intrastrain	Gc e03.04 *n* = 4	Purifying	27	4
*pil*					
	Interstrain	Mc *n* = 71	Purifying	1	100
	Intrastrain	Mc 053442 *n* = 5	Purifying	6	33
	Intrastrain	Mc 8013 *n* = 4	Purifying	8	13
	Intrastrain	Mc alpha14 *n* = 4	Purifying	2	0
	Intrastrain	Mc LNP21362 *n* = 5	Purifying	2	0
	Intrastrain	Mc NZ-05/33 *n* = 11	Purifying	1	100
	Interstrain	Gc *n* = 144	Positive	7	29
	Intrastrain	Gc DGI18 *n* = 5	Purifying	3	100
	Intrastrain	Gc NCCP11945 *n* = 5	Purifying	5	20
	Intrastrain	NG05 *n* = 10	Positive	1	100
	Intrastrain	Gc PID24-1 *n* = 4	Purifying	5	40

Statistically significant *D*_T_ statistics (*p* < 0.05) were used to determine sites under selection, where a positive value of *D*_T_ indicated that the region was under positive selection, and a negative *D*_T_ value signified purifying selection. The genes analyzed are indicated as belonging to either *opa* or *pil*, and analysis performed either within species or strains is also denoted. Strains showing evidence for selection are shown along with the number of *opa* or *pil* genes analyzed. The number of midpoint regions and the percentage of those midpoints that lie within pre-defined constant regions are given.

The PRFMLE program was used to analyze full-length alignments and estimate the per locus mutation rate parameter (μ) and scaled selection pressure (γ). All gene alignments were tested for evidence of interspecies, interstrain (Table 4), and intrastrain (S5 Table) selection pressures along with those that may be dependent on geographical location. By assuming a Poisson distribution, PRFMLE detected evidence of positive selection in all interspecies and interstrain analysis of *pil* and *opa* (Table 4). Interestingly, *opa* and *pil* intrastrain analysis revealed positive selection within the majority of Gc strains (positive selection indicated within ~68% of *pil* and ~44% of *opa*), with less prevalence exhibited within individual strains of Mc (positive selection indicated within ~33% of *pil* and 0% of *opa*) (S5 Table).

**Table 4:** PRFMLE analysis of selection pressures detected from interspecies and interstrain analysis of *pil* and *opa* genes of *N*. *meningitidis* and *N*. *gonorrhoeae*.

Gene	Species	Interpecies or interstrain analysis	γ (95% confidence interval)	Significance (*p* value)	Outcome
*pil n* = 219		Interspecies	3.44 ± 3.11	3.78 x 10^−5^	Positive selection
	*N*. *meningitidis n* = 71	Interstrain	2.47 ± 2.84	6.12 x 10^−3^	Positive selection
	*N*. *gonorrhoeae n* = 148	Interstrain	3.22 ± 3.52	7.73 x 10^−4^	Positive selection
*opa n* = 86		Interspecies	3.44 ± 3.11	3.78 x 10^−5^	Positive selection
	*N*. *meningitidis n* = 17	Interstrain	2.68 ± 2.64	8.67 x 10^−4^	Positive selection
	*N*. *gonorrhoeae n* = 69	Interstrain	3.42 ± 3.36	1.40 x 10^−4^	Positive selection

Investigation into selection pressures using PAML corroborated the PRFMLE data. Two selection models were performed on interspecies alignments of *pil* and *opa* in order to perform a likelihood ratio test (2Δℓ) and compare the null model (*d*_N_/*d*_S_ ≤ 1) with an alternative model (*d*_N_/*d*_S_ > 1). The resulting likelihood values (ℓ) were used to calculate the log likelihood ratio. As the resulting *p*-values of each alignment were less than 0.00001, the null model (*d*_N_/*d*_S_ ≤ 1), which states that polymorphisms arise under neutral conditions, was rejected and the alternative model was presumed to provide a better fit to the observed data. Genes were determined to be under positive selection by the *d*_N_/*d*_S_ ratio of the alignments as established under the alternative model (Table 5). The use of the Bayes Empirical Bayes method was then applied to ascertain the location of positively selected sites in the alignment. This analysis identified the interspecies alignment of *pil*, along with the interstrain alignments of *pil* and *opa* as being under positive selection. While the full-length interspecies alignment of *opa* did not have a *d*_N_/*d*_S_ ratio greater than one, investigation into the site classes revealed that a portion of sites showed evidence for positive selection (~17% had a *d*_N_/*d*_S_ ratio equal to 4.06). The variable minicassettes contained the majority (~97%) of positively selected amino acids within *pil*. In *opa*, the hypervariable domains contained the majority of positively selected amino acids (~75% in hypervariable and ~12% in semivariable) (Fig 7A) [19].

**Table 5:** Likelihood ratio test (LRT) of the selection pressures present in the *pil* and *opa* alignments under the null model (M7) or the alternative model (M8) as implemented in PAML.

Gene	Species	Null model (M7)		Alternative model (M8)		2Δℓ
		ℓ	*d*_N_/*d*_S_	ℓ	*d*_N_/*d*_S_	
*pil n* = 219		-21023.00	0.57	-20027.94	2.58	1,990.12
	*N*. *meningitidis n* = 71	-7558.24	0.64	-7256.25	2.23	603.98
	*N*. *gonorrhoeae n* = 148	-5070.38	0.40	-4732.79	3.09	675.18
*opa n* = 86		-14583.21	0.40	-14174.39	0.94	817.64
	*N*. *meningitidis n* = 17	-3119.45	0.23	-3015.58	1.40	207.74
	*N*. *gonorrhoeae n* = 69	-4316.29	0.40	-4225.30	1.03	181.98

A likelihood ratio greater than $\chi_{1\%}^{2}$ = 9.21 with d.f. = 2 shows statistically significant evidence for the existence of positively selected sites in these genes [64].

The fact that the majority of positively selected sites are harbored within the hypervariable regions of *opa* (Fig 7A) conflicts with previous beliefs that the CEACAM binding domains located within HV1 and HV2 exist in specific combinations that are maintained to conserve receptor binding [86,87]. Therefore, further investigation into the hypervariable regions of Opa was performed to determine if nonsynonymous polymorphisms are biased and show preference for specific types of amino acids (ie. charged, polar, hydrophobic) within the HV1 and HV2 domains. Comparison of the amino acids encoded within the entire Opa alignment with those found within the hypervariable domains can provide insight into any patterns that may be present within these regions. Analysis revealed that the hypervariable domains exhibited variable types of amino acids, with the majority of nonsynonymous sites located within the positively selected regions (Fig 7D and 7E). Not surprisingly, hydrophobic amino acids are less prevalent within both hypervariable regions (analysis of all Opa proteins revealed that, on average, ~22% occurred within the entire protein, ~14% within HV1, and ~16% within HV2) as these regions are exposed to the external environment. However, the positively selected regions within HV2 contain a relatively high amount of these amino acids (~23% hydrophobic aa). Conversely, positively selected regions within HV1 do not display the same propensity for hydrophobic amino acids (~7%), and instead contain a higher percentage of negatively (~10% in HV1 and ~6% in Opa) and positively (~18% in HV1 and ~12% in Opa) charged amino acids compared to the entire Opa protein. Finally, in both the HV1 and HV2 positively selected regions, the quantity of uncharged polar amino acids (~27% in HV1 and ~28% in HV2) are higher than those found in the entire protein (~15%). Therefore, positive selection within both HV1 and HV2 differs, as amino acids within these regions are distinct from the entire protein and are additionally varied from the HV regions that were not determined to be under the influence of positive selection.

Solvent accessible surface analysis performed on the Opa 60 protein of strain MS11 within the PDB databank [75] revealed that relatively few amino acids are predicted to buried (only ~26% of all amino acids) with an average solvent-accessible surface area ratio <20%. However, the majority of these buried amino acids are hydrophobic (~27% of all buried amino acids), with the membrane spanning regions (~37%) and the hypervariable domains (~5% in HV1 and ~10% in HV2) containing nearly half of the buried hydrophobic amino acids. Conversely, about half of the amino acids in Opa are predicted to be solvent exposed (~42% of all amino acids) with an average solvent-accessible surface area ratio >50%. Interestingly, when these positions were analyzed against the entire Opa protein alignment, the majority of these solvent-exposed amino acids are hydrophobic (~42% of all types of amino acids are hydrophobic). However, as would be expected, a little over half of the hydrophobic solvent-exposed amino acids are found within membrane spanning regions (~55%). Nonetheless, the hypervariable domains also contain a relatively large amount of solvent-exposed hydrophobic amino acids, with a little less than half of them predicted to be under positive selection as determined by PAML (~11% present within HV1, ~20% of which are under positive selection, and ~17% within HV2, ~52% of which are under positive selection) (S6 Table).

### Structural prediction of selected sites

**Fig 8:**
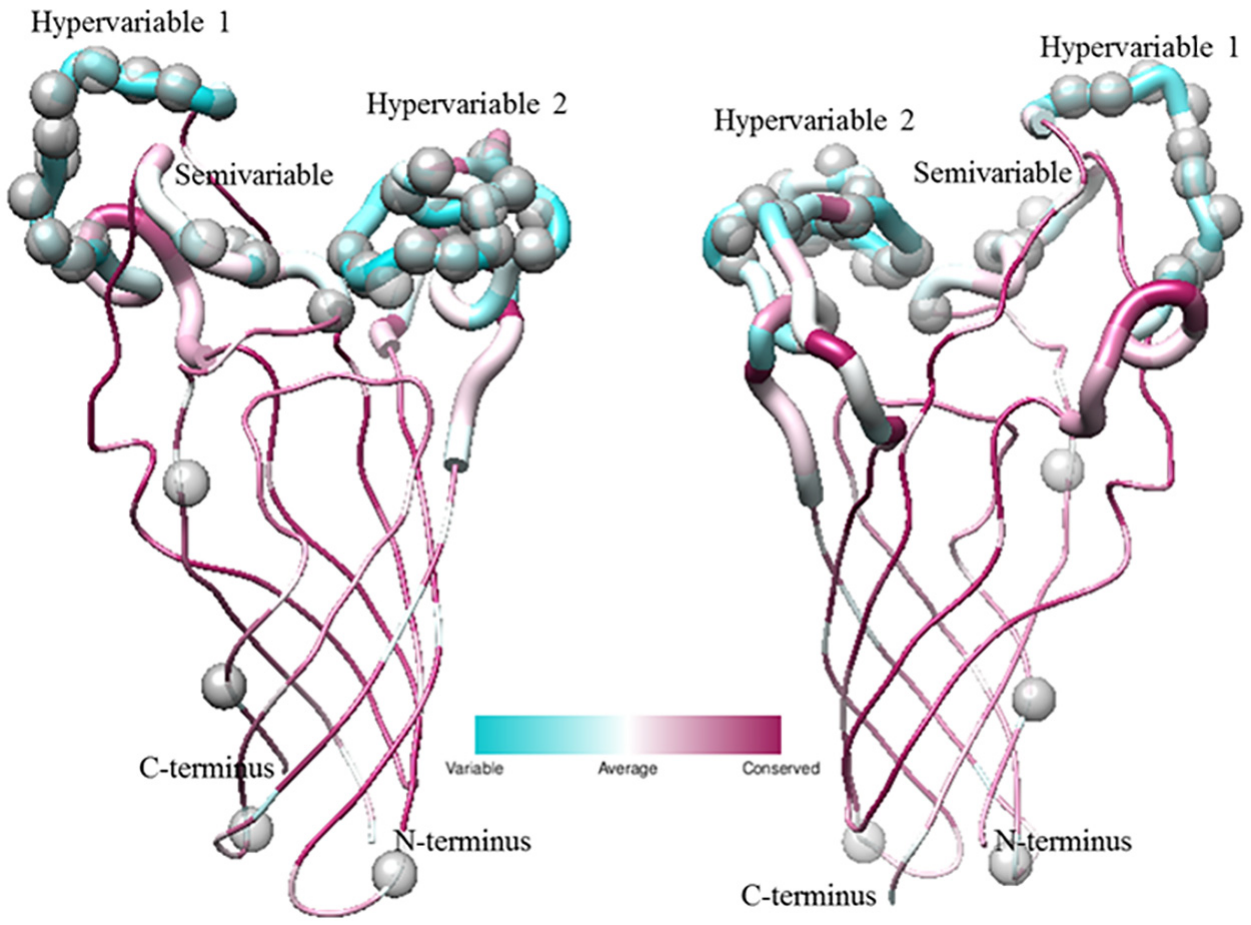
Location of positively selected amino acids in Opa. The three dimensional structure of Opa was obtained from PDB ID 2MAF [75]. The Consurf Server was used to align the amino acid multiple sequence alignment of the Opa proteins used in this study to the sequence of the 2MAF Opa60 structure [71–74]. Polymorphisms present in the Opa proteins from this study were depicted with UCSF Chimera, where cyan sites indicate the highest degree of variability and maroon sites indicate the most conserved regions [76]. The ribbons denoting the SV, HV1, and HV2 regions are shown larger than the other areas of the protein. The positively selected sites determined from the Bayes Empirical Bayes theorem implemented through PAML are shown as gray spheres [46]. This analysis shows that the three variable regions of the protein show strong evidence for being under positive selection, with the conserved regions showing relatively no evidence of sequence diversity or selection.

**Fig 9:**
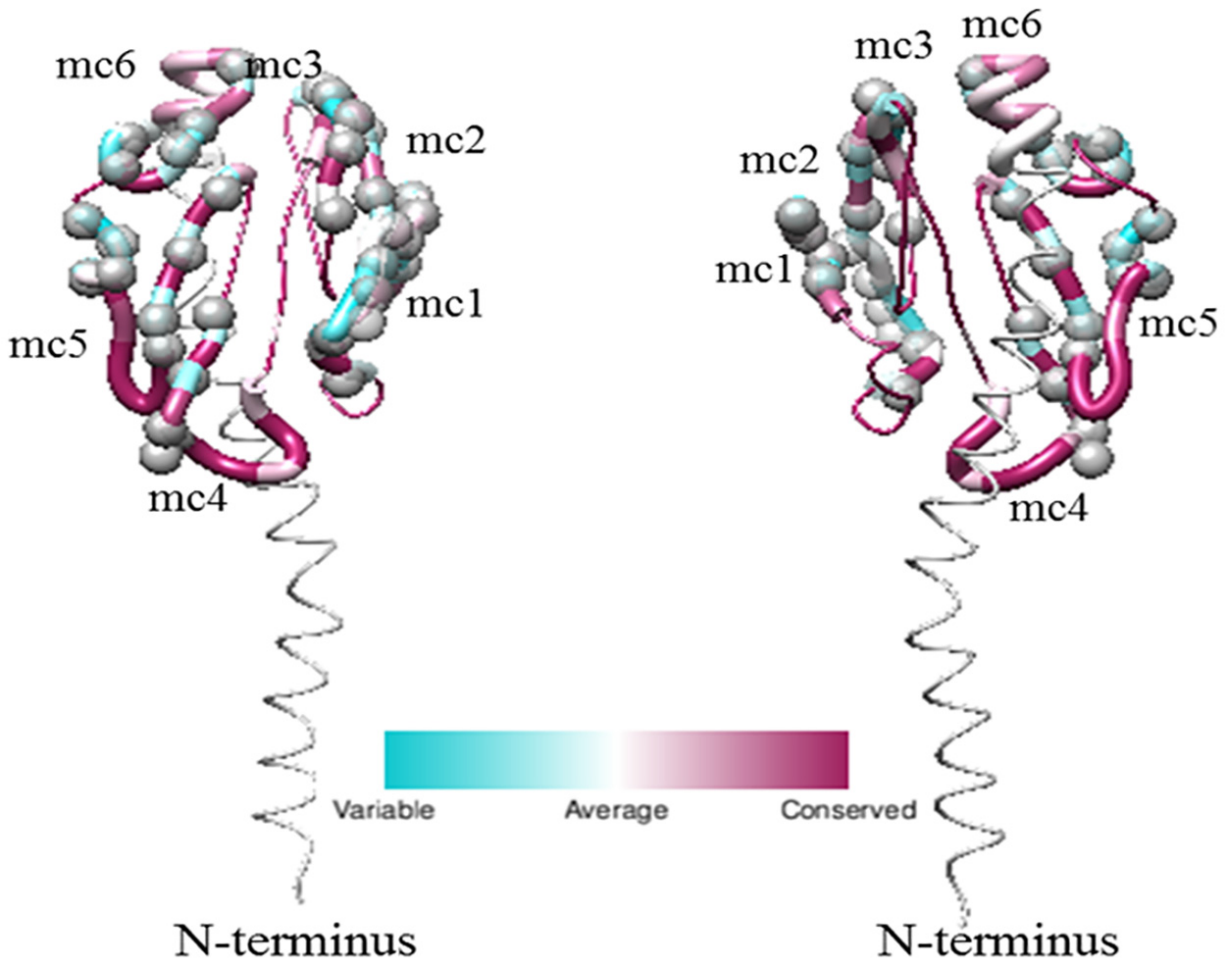
Location of positively selected amino acids in Pil. The three dimensional structure of Pil was obtained from PDB ID 2HIL [15]. The Consurf Server was used to align the amino acid multiple sequence alignment of the Pil proteins used in this study to the sequence of the 2HIL structure [71–74]. Polymorphisms present in the Pil proteins from this study were depicted with UCSF Chimera, where black sites indicate the highest degree of variability and white sites indicate the most conserved regions [76]. The ribbons denoting the variable minicassettes are shown larger than the other areas of the protein. The positively selected sites determined from the Bayes Empirical Bayes theorem implemented through PAML are shown as grey spheres [46]. This analysis shows that the variable regions of the protein show strong evidence for being under positive selection, with the conserved regions showing relatively no evidence of sequence diversity or selection.

In order to visualize the location of sites predicted to be under the influence of selection, multiple sequence alignments were used to construct 3D protein assemblies via experimentally determined structures available through PDB. This provided further insights into the location of amino acids predicted to be under the influence of positive selection and into the forces acting on these virulence genes among species and strains. The majority of positively selected sites within Opa (Fig 8) were located within the variable regions SV, HV1, and HV2 [19,23,24,75]. Solvent accessible surface area analysis revealed that a little over half of these positively selected sites (~52%) lie within solvent-exposed regions, while the majority of the remaining positively selected sites contain variable surface-exposed ratios (~38% contain ratios between 20–50%) indicating that their surface-exposure remain undetermined (S6 Table). Corresponding to previous data [24], the more constant membrane spanning and periplasmic regions contained several positively selected sites. All sites determined to be under positive selection within PilE proteins (Fig 9) lay within the pre-determined variable regions [13]. Further solvent accessible surface area analysis of these sites revealed that a little over half of the positively selected sites (~54%) are predicted to be solvent exposed. However, similar to Opa, the majority of remaining amino acids contain solvent-exposed surface areas between those of buried and solvent-exposed amino acids (~36% contain ratios between 20–50%) (S7 Table).

## Discussion

The maintenance of multiple, variable gene copies that encode a single antigenic determinant within pathogenic bacteria is believed to facilitate immune evasion. The evolution and maintenance of these variable genes has been attributed to inter- or intragenic transfer of alleles [7,87,88], mutation [81], or a combination of these driven by diversifying selection [81,88]. Previous analyses of closely related *opa* sequences (11 *opa* gene sequences from 14 *N*. *gonorrhoeae* strains) have attributed the majority of sequence polymorphisms to intragenic recombination among existing alleles with little evidence to support the horizontal transfer of genes among strains, even within mixed gonococcal infections [87]. A separate analysis utilizing the same dataset of *N*. *gonorrhoeae opa* genes, in addition to *N*. *meningitidis opa* alleles (297 *opa* gene sequences from pathogenic and nonpathogenic strains of *N*. *meningitidis*; [86]) determined that Mc *opa* genes are distinct from Gc *opa* genes as they typically harbor genetically identical alleles [89]. Consequently, this implies that host immune pressures act to suppress the number of unique combinations of HV1 and HV2 regions in Mc Opa variants [86], but increase the number and diversity of *opa* alleles in Gc [89]. Similarly, investigation into *pil* sequence polymorphisms has been limited to only two *N*. *meningitidis* strains (1 *pilE* and 8 *pilS* sequences from strain Z2491 and 1 *pilE* and 8 *pilS* sequences from strain MC58) and provided strong evidence for sequence polymorphisms arising via intragenic recombination and horizontal gene transfer [81]. However, to date, no comprehensive analysis of sequence polymorphisms and selection pressures within the variable *opa* and *pil* genes of multiple species and strains of pathogenic *Neisseria* from distinct geographical locations has been determined. Therefore, this study sought to clarify the degree of genetic variability and deduce the presence of selective pressures acting upon available *opa* and *pil* sequences.

A proposed mechanism to describe the genetic variability displayed within the *opa* [20] and *pil* [81,82] alleles implicates horizontal gene transfer and subsequent recombination of *opa* and *pil* genes. While statistically significant horizontal transfer events of *opa* and *pil* genes among and between species and strains was detected (Fig 6), only intrastrain genetic transfer could account for the polymorphisms detected within the majority of strains determined to have significant D′ values (within ~88% of *opa*-encoding strains and ~97% of *pil*-encoding strains) (Table 2 and S3 and S4 Tables). Therefore, the preponderance of polymorphisms is attributable to linkage equilibrium within strains. Additionally, phylogenetic analysis revealed that divergence of these variable genes is only dependent upon the species, as the vast majority of *opa* and *pil* genes contain polymorphisms, insertions and deletions that are unique to either *N*. *meningitidis* or *N*. *gonorrhoeae*, and display no predisposition towards disease severity (ring 4 in Fig 1), serogroup (ring 4 in Fig 1), or geographical distribution (ring 3 in Fig 1) (as indicated by the solid lines above the tree in Fig 1). Consequently, this implies that, in addition to having diverged prior to geographical distribution, these genes are not linked to other virulence determinants and only intrastrain horizontal gene transfer events are evident in variant forms of these alleles.

While homologous recombination appears to account for the majority of polymorphisms detected within the variable genes of pathogenic *Neisseriae*, selection pressures have been presumed to drive antigenic variation. Efforts were taken to extrapolate whether outside forces or the intrinsic tendency for recombination and purifying selection of deleterious mutations drives the observed polymorphisms. Initial investigation indicated a role for positive selection in both the interspecies analysis of *pil* and *opa* and the Mc interstrain analysis of *pil* and *opa* as the *d*_N_/*d*_S_ ratios were greater than one (Table 1). However, interstrain analysis of Gc *opa* genes did not show evidence for positive selection as the *d*_N_/*d*_S_ ratio was less than one (Table 1). This is rather surprising as previous analysis revealed that *N*. *meningitidis opa* alleles were less diverse than those isolated from *N*. *gonorrhoeae*, indicating strong positive selection in the latter [89]. However, while these conclusions were drawn from a robust sample of *N*. *meningitidis* (297 sequences) and *N*. *gonorrhoeae* (154 sequences) *opa* alleles, each species was acquired from a single geographic location (the Czech Republic [86] for *N*. *meningitidis* and Sheffield and London, England for *N*. *gonorrhoeae* [87]). In contrast, the data used in this study utilized a more diverse sample of *opa* genes as six *N*. *meningitidis* and sixteen *N*. *gonorrhoeae* strains from distinct geographic locations (including China, France, Germany, and Africa for *N*. *meningitidis* and Sweden, North America, and Russia for *N*. *gonorrhoae*) were sampled. Therefore, while phylogenetic analysis indicated that the presence of polymorphisms was not linked to geographical isolation of strains (Fig 1), the more diverse data set used in this analysis may have provided a larger array of polymorphisms yielding a more robust *d*_N_ statistic.

To further extrapolate sites under selective pressures, a sliding window approach was implemented. This analysis revealed evidence for positive selection within interspecies and interstrain *pil* genes (Fig 7b), while intrastrain analysis of *opa* and *pil* detected purifying selection within several strains (Table 3). The absence of positively selected regions within a single strain as compared to their presence among species and strains indicates a greater divergence of *pil* genes in the latter categories (interspecies and interstrain). Such divergence is evidenced in the greater number of nonsynonymous polymorphisms between strains and species resulting in a significant *D*_T_ statistic (*p* < 0.05) that indicates positive selection as the driving force behind this divergence (Fig 7b). The negatively selected sites detected from the intrastrain analysis of *pil* may similarly be masked in the interspecies and interstrain analysis by the high degree of sequence polymorphisms (Table 1 and Fig 2b). While purifying selection is presumed to occur within constant regions to conserve necessary functional and/or structural elements for the encoded proteins, only a portion of sites under intrastrain purifying selection within both *pil* and *opa* occurred within predefined invariable regions. Due to this, it may be speculated that variable regions under purifying selection also encode structural elements for the encoded proteins. Furthermore, although identified through a codon substitution model, a portion of the variable minicassettes containing negatively selected sites within *pil* share sequences homologous to experimentally verified non-canonical internal promoter elements [83,85]. Consequently, further investigation into a small RNA transcriptome of Gc strain MS11 revealed the presence of two non-canonical promoter motifs upstream of *pil*-derived sRNAs which correspond, in sequence, to experimentally verified *pilE* and *pilS* promoters (data not shown; [83,85]). Investigation into their conservation revealed that these promoter motifs are maintained as highly conserved elements (100% conservation of motif 1 and ~97% conservation of motif 2) within all species and strains of pathogenic *Neisseria* analyzed regardless of their location in predefined invariable (motif 1 in mc5) or constant regions (motif 2) (Fig 7C). Therefore, these regions may be placed under negative selection to maintain the internal promoters. Additionally, with regard to Opa, as CEACAM binding by Opa is determined by the hypervariable domains [25–27], and, as negative selection for CEACAM binding has been previously determined *in vivo* [90], it is likely that these cells are maintaining a preferred binding potential as the negatively selected sites within the variable regions of *opa* occurred within the hypervariable domains as previously speculated [6].

Additional investigation into selective pressures with PRFMLE identified evidence for positive selection in all interspecies and interstrain analysis, which were confirmed in interspecies *pil* and interstrain *opa* and *pil* by PAML, as these analyses determined that the *d*_N_/*d*_S_ ratios were greater than one (Tables 4 and 5). While full-length interspecies analysis of *opa* by PAML did not reveal evidence for positive selection, further differentiation revealed that a portion of the interspecies *opa* sequence (~17%) contained a *d*_N_/*d*_S_ ratio greater than one (*d*_N_/*d*_S_ = 4.06). While these tests of neutrality differ on the extent of selection within the *opa* and *pil* variable genes of *Neisseria*, both conclude that portions of these genes are under the influence of positive selection. The statistically significant positively selected sites determined through a Bayes Empirical Bayes approach were primarily located within the exposed variable domains of Opa and PilE (Fig 7 and S2 and S3 Figs). The existence of positively selected sites within the *pil* conserved (detected within ~6% of sites under positive selection) and semivariable (detected within ~30% of sites under positive selection) regions was unanticipated, as previous analysis of PilE only detected positive selection within the hypervariable domains [81]. However, these sites were obtained from investigation of 18 *pil* sequences from two *N*. *meningitidis* strains (*N*. *meningitidis* Z2491 and MC58), while this study analyzed 219 individual *pil* genes from multiple strains of *N*. *meningitidis* and *N*. *gonorrhoeae*. Similarly, the high degree of positively selected sites within the hypervariable domains of *opa* (detected within ~71% of sites under positive selection) conflicts with previous beliefs that the CEACAM binding domains located within HV1 and HV2 exist in specific combinations that are maintained to conserve receptor binding [86,87]. Indeed, a high degree of amino acid variability was found to exist within these positively selected hypervariable regions with relatively little evidence for any pattern associated with the polymorphisms (Fig 7D and 7E). While both HV1 and HV2 contain fewer hydrophobic residues than those found in the entire alignment (~14% of amino acids in HV1, ~16% of amino acids in HV2, vs ~22% in Opa), the regions determined to be under positive selection within HV2 contain a substantially higher proportion of hydrophobic amino acids (~23%). Furthermore, as many of the predicted solvent-exposed hydrophobic amino acids within HV2 were also determined to be under the influence of positive selection (~52%), tThis apparent selection for hydrophobic residues may aid in CEACAM binding. This is further supported as previous analysis of CEACAM1 identified critical roles for hydrophobic amino acid residues in Opa interactions [91,92]. The lack of preference for hydrophobic amino acids within the selected regions of HV1 (only 20% of the solvent-exposed hydrophobic amino acids) and the tendency for this region to contain a higher amount of charged or polar amino acids (~10% negatively charged, ~18% positively charged, ~27% uncharged polar) than what is found in the entire protein alignment (~6% negatively charged, ~12% positively charged, ~15% uncharged polar) does not appear to confer an advantage in CEACAM binding as evidence suggests hydrophobic interactions are also involved in receptor binding for this domain [91,92]. Other host adhesion receptors, however, contain hydrophilic interfaces with a significant number of charged residues involved in receptor binding (such as CD2 and CD58), therefore if the selected regions in HV1 are involved in receptor binding they may confer differential receptor specificity [92]. Consequently, this analysis was able to further extrapolate selected sites between available Opa and Pil encoding genes among a multitude of *N*. *meningitidis* and *N*. *gonorrhoeae* species and strains.

In conclusion, analysis of variable alleles within pathogenic *Neisseria* has allowed insights into the evolution and maintenance of the *opa* and *pil* genes across geographical locations, species and strains. Phylogenetic and gene conversion analysis have indicated that homologous recombination via horizontal gene transfer within strains is the most likely source of novel polymorphisms (S3 and S4 Tables). Since there is evidence for horizontal exchange of *opa* and *pil* between and within species (Fig 6), the fact that statistically significant horizontal transfer events were only detected within intrastrain polymorphisms should be further elucidated, perhaps through more sensitive tests for linkage equilibrium. However, it is likely that physical separation due to the typical sites of infection for *N*. *meningitidis* and *N*. *gonorrhoeae* has not allowed for significant interspecies horizontal exchange of *pil* and *opa* genes, a factor which has most likely played a role in the divergence of these genes between *N*. *meningitidis* and *N*. *gonorrhoeae* (Fig 1). Regardless, selection pressures applied by the host immune system appear to be the catalyst for these gene conversion events as the majority of polymorphisms are nonsynonymous causing *d*_N_/*d*_S_ ratios to be greater than one (Table 1). While the variable portions of these genes contained the majority of positively selected sites (Fig 7), the degree of positive selection in these regions was great enough to skew the *d*_N_/*d*_S_ ratio of the entire gene in favor of positive selection (Table 1) despite evidence for purifying selection in certain regions of the genes (Table 3). Therefore, due to the large quantity of nonsynonymous polymorphisms located mainly within exposed variable portions of the encoded peptides (Figs 3, 4 and 7), strong positive selection pressures were detected within these variable regions of *opa* and *pil* (Tables 1, 4 and 5). Indeed, the occurrence of positive selected surface exposed regions is not unique to *Neisseria*, as this has also been observed in vampire bat venom, and has led to the theorization that these polymorphisms act to oppose the immunological resistance developed in prey animals [93]. Therefore, within *Neisseria*, these strong positive pressures correspond to the obligate pathogenic nature of this pathogen, as it undergoes persistent scrutiny from the host immune system.

## Supporting Information

S1 Data *Neisseria opa* gene sequences utilized in this analysis. (TXT)

S2 Data *Neisseria pil* gene sequences utilized in this analysis. (TXT)

S1 Fig Sequence logo of *Neisseria opa* and *pil* alignments used in this analysis. The sequence logo of *opa* (*n* = 86) includes only the hypervariable domains and spans from base pair 397–891 of the alignment (a). The sequence logo of *pil* (*n* = 219) only includes the variable minicassettes and spans from base pair 1–495 in the alignment. The overall height of the nucleotide or amino acid indicates the sequence conservation in the alignment at that position. The height of nucleotides or amino acids within a stack indicates the relative frequency of each at that position.
(TIF)

S2 Fig *pil p*hylogenetic trees. 219 *pil* genes analyzed with (a) Fasttree discrete gamma model, (b) PhyML 3 HKY85, or (c) GTR models. The colored boxes closest to the trees indicate the strain of *N*. *gonorrhoeae* or *N*. *meningitidis* (ring 1), while the second ring signifies the species of the isolate, either *N*. *gonorrhoeae* or *N*. *meningitidis*. The third ring denotes the geographical region of the isolate, when known, while the outermost ring indicates the severity of the disease or the serogroup of the strain. The bootstrap values as calculated with the Shimodaira-Hasegawa test are depicted as colored branches, where red branches indicate minimum bootstrap values (0), green indicate median bootstrap values, and blue indicate maximum bootstrap values (1).
(TIF)

S3 Fig *opa p*hylogenetic trees. 86 *opa* genes analyzed with (a) Fasttree discrete gamma model, (b) PhyML 3 HKY85, or (c) GTR models. The colored boxes closest to the trees indicate the strain of *N*. *gonorrhoeae* or *N*. *meningitidis* (ring 1), while the second ring signifies the species of the isolate, either *N*. *gonorrhoeae* or *N*. *meningitidis*. The third ring denotes the geographical region of the isolate, when known, while the outermost ring indicates the severity of the disease or the serogroup of the strain. The bootstrap values as calculated with the Shimodaira-Hasegawa test are depicted as colored branches, where red branches indicate minimum bootstrap values (0), green indicate median bootstrap values, and blue indicate maximum bootstrap values (1).
(TIF)

S1 Table The *opa* and *pil* genes utilized in this study. The species and strain of *Neisseria* is given along with the Genbank accession number, locus tag, and the chromosomal location of the gene. In some instances, the gene was lengthened to include important sequence features or shortened to remove repeat sequence elements; in either case the genomic location of the gene used in this paper is shown.
(DOCX)

S2 Table Test of substitution saturation of the *opa* and *pil* nucleotide alignments. (DOCX)

S3 Table Evidence of linkage equilibrium within the *opa* genes of *N*. *gonorrhoeae* and *N*. *meningitidis* species. (DOCX)

S4 Table Evidence of linkage equilibrium within the *pil* genes of *N*. *gonorrhoeae* and *N*. *meningitidis* species. (DOCX)

S5 Table PRFMLE analysis of variable genes within individual strains of Mc and Gc. Genes are shown along with the strain of Mc or Gc and the number of sequences that were analyzed. The value of γ and the *p* value for that estimate are also shown.
(DOCX)

S6 Table Proportion of amino acids in Opa predicted to be buried or solvent exposed. (DOCX)

S7 Table Proportion of amino acids in Pil predicted to be buried or solvent exposed. (DOCX)
