# Supplementary figures and images for: Positive Selection Pressure Drives Variation on the Surface-Exposed Variable Proteins of the Pathogenic Neisseria
Source: PLoS One. 2016 Aug 17;11(8):e0161348. doi: 10.1371/journal.pone.0161348 (PMC5020929; doi:10.1371/journal.pone.0161348)

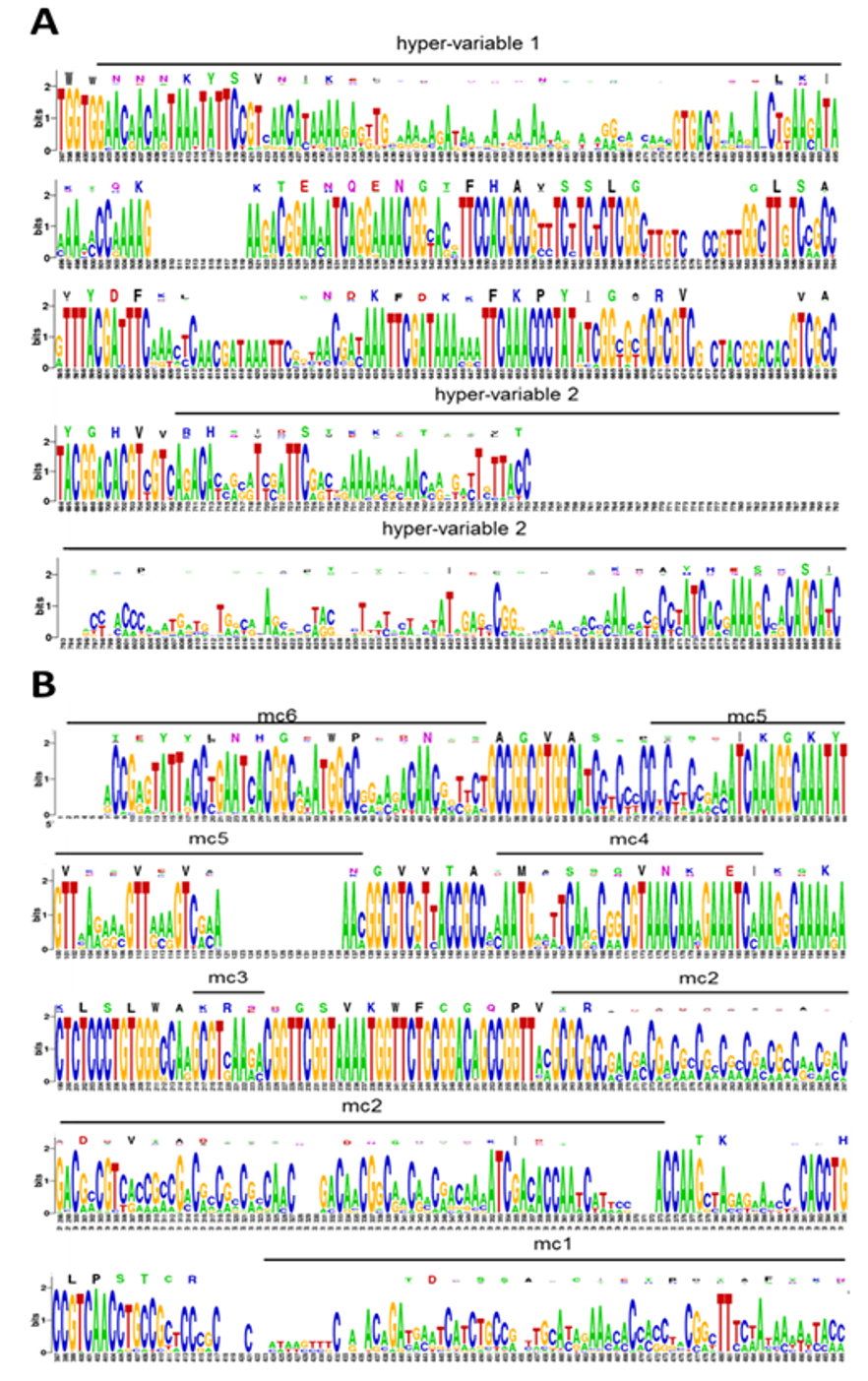

Supplement: S1 Fig — The sequence logo of opa (n = 86) includes only the hypervariable domains and spans from base pair 397–891 of the alignment (a). The sequence logo of pil (n = 219) only includes the variable minicassettes and spans from base pair 1–495 in the alignment. The overall height of the nucleotide or amino acid indicates the sequence conservation in the alignment at that position. The height of nucleotides or amino acids within a stack indicates the relative frequency of each at that position. (TIF) [file pone.0161348.s003.tif]

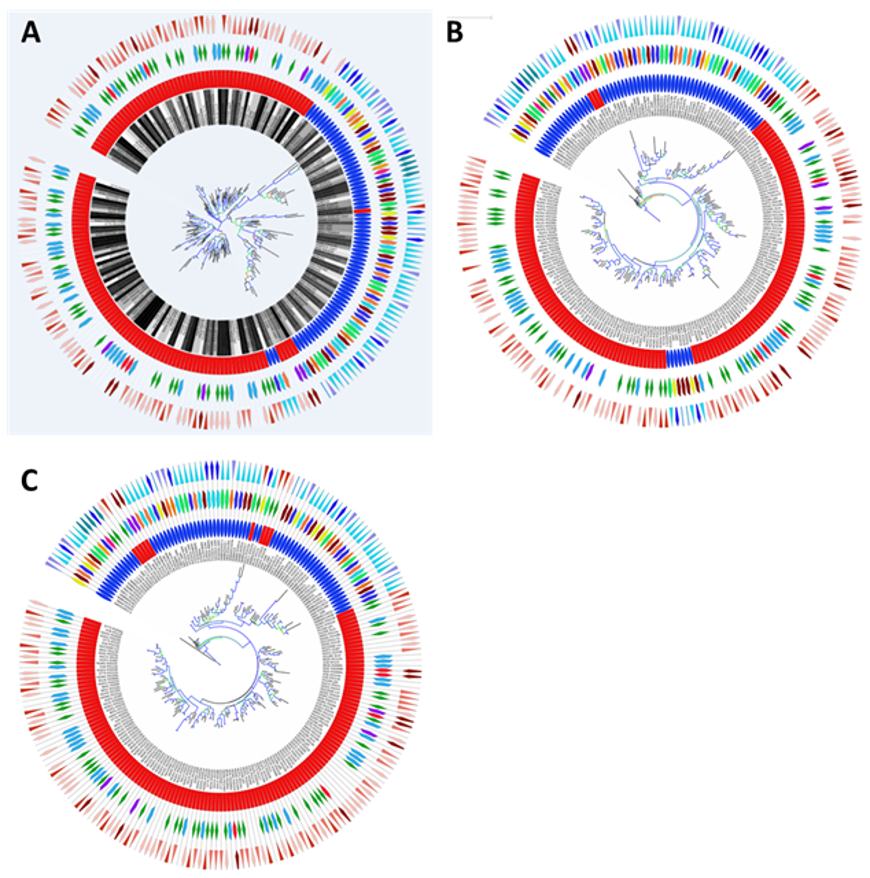

Supplement: S2 Fig — 219 pil genes analyzed with (a) Fasttree discrete gamma model, (b) PhyML 3 HKY85, or (c) GTR models. The colored boxes closest to the trees indicate the strain of N. gonorrhoeae or N. meningitidis (ring 1), while the second ring signifies the species of the isolate, either N. gonorrhoeae or N. meningitidis. The third ring denotes the geographical region of the isolate, when known, while the outermost ring indicates the severity of the disease or the serogroup of the strain. The bootstrap values as calculated with the Shimodaira-Hasegawa test are depicted as colored branches, where red branches indicate minimum bootstrap values (0), green indicate median bootstrap values, and blue indicate maximum bootstrap values (1). (TIF) [file pone.0161348.s004.tif]

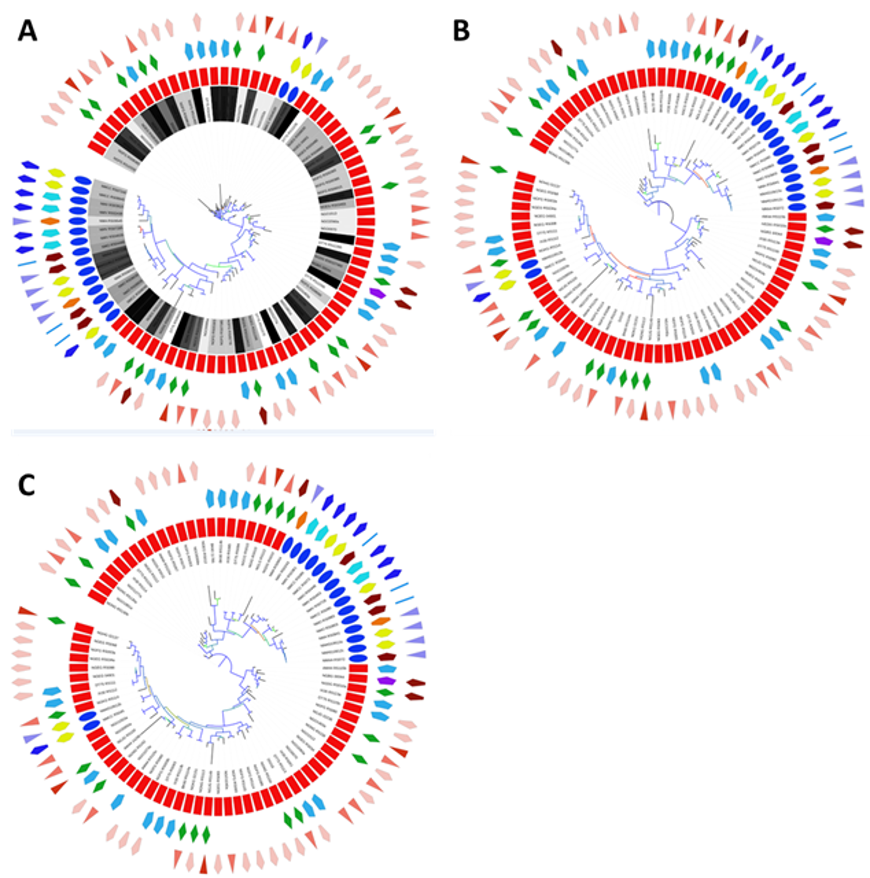

Supplement: S3 Fig — 86 opa genes analyzed with (a) Fasttree discrete gamma model, (b) PhyML 3 HKY85, or (c) GTR models. The colored boxes closest to the trees indicate the strain of N. gonorrhoeae or N. meningitidis (ring 1), while the second ring signifies the species of the isolate, either N. gonorrhoeae or N. meningitidis. The third ring denotes the geographical region of the isolate, when known, while the outermost ring indicates the severity of the disease or the serogroup of the strain. The bootstrap values as calculated with the Shimodaira-Hasegawa test are depicted as colored branches, where red branches indicate minimum bootstrap values (0), green indicate median bootstrap values, and blue indicate maximum bootstrap values (1). (TIF) [file pone.0161348.s005.tif]
