# Supplementary material for: Positive Selection Pressure Drives Variation on the Surface-Exposed Variable Proteins of the Pathogenic Neisseria
Source: PLoS One. 2016 Aug 17;11(8):e0161348. doi: 10.1371/journal.pone.0161348 (PMC5020929; doi:10.1371/journal.pone.0161348)
Supplement: S1 Table — The species and strain of Neisseria is given along with the Genbank accession number, locus tag, and the chromosomal location of the gene. In some instances, the gene was lengthened to include important sequence features or shortened to remove repeat sequence elements; in either case the genomic location of the gene used in this paper is shown. (DOCX) [file pone.0161348.s006.docx]

**S1 Supplemental Table**

| Strain | Genbank Accession | Locus tag | Annotated location | Paper location | Annotation |
| --- | --- | --- | --- | --- | --- |
| *N. gonorrhoeae* 3502 | NZ_DS999992.1 | NGBG_RS107625 | 67634-68038 | 67634-68038 | large pilS cassette protein |
|  |  | NGBG_RS107625 | 67634-68038 | 67621-68038 | large pilS cassette protein |
|  |  | NGBG_RS107625 | 67634-68038 | 68147-68531 | large pilS cassette protein |
|  |  | NGBG_RS107535 | 95257-95613 | 95263-95597 | hypothetical protein |
|  |  | NGBG_RS107535 | 95257-95613 | 95598-95918 | hypothetical protein |
|  |  | NGBG_RS107610 | 72079-72447 | 72079-72586 | pilin |
|  |  | NGBG_00044 | 47212-47928 | 47165-47928 | outer membrane protein |
| *N. gonorrhoeae* DGI2 | NZ_GG749039.1 | NGMG_RS09945 | c24753-25106 | c24749-25138 | hypothetical protein |
|  | NZ_GG749050.1 | NGMG_RS0106660 | 599539-600105 | 600146-600544 | large pilS cassette |
|  |  | NGMG_RS16065 | 574991-575578 | 574978-575407 | large pilS cassette |
|  |  | NGMG_RS16160 | 597989-598363 | 597957-598347 | PilE/Pilin |
|  |  | NGMG_RS16050 | 570992-571435 | 570949-571302 | hypothetical protein |
|  |  | NGMG_RS0106630 | 571327-571705 | 571303-571649 | hypothetical protein |
|  |  | NGMG_RS16165 | 597989-598363 | 598348-598694 | PilE/Pilin |
|  |  | NGMG_RS0106660 | 599539-600105 | 599178-599410 | large pilS cassette |
|  |  | NGMG_RS0106660 | 599539-600105 | 600545-601019 | large pilS cassette |
| *N. gonorrhoeae* DGI18 | NZ_EQ972648.1 | NGCG_RS0100005 | c266-1007 | c266-1006 | protein II |
|  | NZ_EQ972649.1 | NGCG_00143 | c138734-139168 | c138881-139222 | fimbrial protein MS11-D1 |
|  |  | NGCG_00142 | c138328-138618 | c138236-138681 | large pilS cassette |
|  |  | Unannotated |  | c341005-341611 |  |
|  | NZ_EQ972673.1 | NGCG_01939 | 7308-7682 | 7276-7682 | pilin |
|  |  | NGCG_RS0107700 | 7308-7682 | 7319-7682 | pilin |
|  | NZ_EQ972688.1 | NGCG_RS0111055 | c98-493 | c98-487 | pilin family protein |
| *N. gonorrhoeae* e0304 | NZ_JFAZ01000037.1 | Unannotated |  | 19050-19497 | BLAST |
|  | NZ_JFAZ01000068.1 | AW44_RS11165 | 1373-1747 | 1341-1868 | hypothetical protein |
|  |  | AW44_RS11170 | 1880-2245 | 1869-2566 | hypothetical protein |
|  | NZ_JFAZ01000083.1 | AW44_14290 | c762-1420 | c762-1394 | hypothetical protein |
|  | NZ_JFAZ01000088.1 | AW44_RS11510 | 1-767 | 15-767 | opacity protein Opa54 |
|  | NZ_JFAZ01000090.1 | AW44_RS11525 | c1-730 | c306-743 | large pilS cassette |
|  | NZ_JFAZ01000093.1 | AW44_RS11545 | 54-767 | 6-767 | opacity-associated protein |
|  | NZ_JFAZ01000096.1 | AW44_RS11570 | c39-639 | c39-639 | opacity-associated protein |
|  | NZ_JFAZ01000097.1 | AW44_RS11575 | 298-627 | 213-627 | hypothetical protein |
|  | NZ_JFAZ01000111.1 | AW44_RS11640 | c25-362 | c25-362 | hypothetical protein |
| *N. gonorrhoeae* F62 | NZ_GG749348.1 | NGNG_RS0105355 | 367022-367387 | 366594-366995 | L-pilin |
|  |  | NGNG_RS0105355 | 367022-367387 | 366996-367387 | L-pilin |
|  |  | NGNG_RS0105325 | 343497-343916 | 343535-344040 | pilin |
|  | NZ_GG749356.1 | NGNG_RS0105700 | c445-834 | c415-888 | hypothetical protein |
|  | NZ_GG749357.1 | NGNG_RS10800 | c77-814 | c1-409 | large pilS cassette protein |
|  |  | NGNG_RS10800 | c77-814 | c410-808 | large pilS cassette protein |
|  | NZ_GG749358.1 | NGNG_RS10810 | c253-999 | c253-604 | large pilS cassette protein |
|  |  | NGNG_RS10810 | c253-999 | c637-1031 | large pilS cassette protein |
|  | NZ_GG749359.1 | NGNG_RS0105705 | c1-291 | c83-423 | hypothetical protein |
|  | NZ_GG749374.1 | NGNG_RS11370 | 50-724 | 2-724 | opacity-associated protein |
|  | NZ_GG749379.1 | NGNG_RS13080 | c38-684 | c38-684 | opacity-associated protein |
|  | NZ_GG749380.1 | NGNG_RS13085 | c1-686 | c1-653 | opacity protein opA52 |
|  | NZ_GG749381.1 | NGNG_RS13095 | c570-929 | c431-917 | fimbrial protein |
|  | NZ_GG749389.1 | NGNG_RS15435 | 426069-426755 | 426021-426755 | opacity-associated protein |
|  |  | NGNG_RS15830 | c501797-502507 | c501797-502556 | opacity-associated protein |
|  | NZ_GG749392.1 | NGNG_RS16255 | c126-821 | c126-821 | hypothetical protein |
| *N. gonorrhoeae* FA1090 | NC_002946.2 | NGO2068 | c2046714-2047403 | c2042801-2043147 | membrane protein |
|  |  | NGO2041a | c2015482-2015907 | c2015343-2015868 | pilin |
|  |  | NGO2068 | c2046714-2047403 | c2040723-2041203 | membrane protein |
|  |  | NGO2068 | c2046714-2047403 | c2041646-2042026 | membrane protein |
|  |  | Unannotated |  | c2045843-2046256 |  |
|  |  | NGO2068 | c2046714-2047403 | c2043148-2043522 | membrane protein |
|  |  | Unannotated |  | c2044392-2044736 |  |
|  |  | NGO2041a | c2015482-2015907 | c2016707-2017060 | pilin |
|  |  | Unannotated |  | c2044773-2045127 |  |
|  |  | Unannotated |  | c2045506-2045842 |  |
|  |  | Unannotated |  | c2037383-2038021 |  |
|  |  | NGO2041a | c2015482-2015907 | c2016302-2016669 | pilin |
|  |  | NGO1512 | c1479533-1480408 | 1480693-1481248 | membrane protein |
|  |  | NGO2068 | c2046714-2047403 | c2042470-2042800 | membrane protein |
|  |  | NGO2068 | c2046714-2047403 | c2041256-2041603 | membrane protein |
|  |  | Unannotated |  | c2014626-2015018 |  |
|  |  | Unannotated |  | c2043862-2044354 |  |
|  |  | Unannotated |  | c2013715-2014201 |  |
|  |  | Unannotated |  | c2014238-2014567 |  |
|  |  | NGO0950a | 925084-925782 | 925084-925782 | opacity protein |
|  |  | NGO1073a | 1035309-1035998 | 1035309-1035998 | opacity protein |
|  |  | NGO1553a | 1531422-1532120 | 1531422-1532120 | opacity protein |
|  |  | NGO1040a | c999760-1000440 | c999760-1000440 | opacity protein |
|  |  | NGO0070 | c74783-75580 | c74783-75580 | opacity protein opA58 |
|  |  | NGO2060a | c2036384-2037067 | c2036384-2037067 | opacity protein |
|  |  | NGO1513 | 1481445-1482281 | 1481445-1482281 | opacity protein OpaD |
|  |  | NGO1463a | c1427614-1428327 | c1427614-1428327 | opacity protein |
|  |  | NGO1277a | 1231620-1232324 | 1231620-1232324 | opacity protein |
|  |  | NGO1861a | c1830206-1830913 | c1830206-1830913 | opacity protein |
|  |  | NGO0066a | c69021-69710 | c69021-69710 | opacity protein |
| N. gonorrhoaee FA19 | NZ_KI391931.1 | NGEG_RS03480 | c631301-631987 | c631301-632035 | opacity-associated protein |
|  |  | NGEG_RS08830 | c1617720-1618409 | c1617720-1618457 | opacity-associated protein |
|  |  | NGEG_RS02290 | c414796-415575 | c413867-414377 | large pilS cassette |
|  |  | NGEG_RS02480 | c446067-446402 | c445534-445975 | pilS cassette |
|  |  | NGEG_RS02430 | c439132-439539 | c439010-439501 | pilS cassette |
|  |  | NGEG_RS02500 | c448491-449171 | c448352-448832 | fimbrial protein |
|  |  | NGEG_RS02485 | c446441-446821 | c446457-446846 | pilS cassette |
|  |  | NGEG_RS02290 | c414796-415575 | c415169-415607 | large pilS cassette |
|  |  | NGEG_RS02440 | c441060-441431 | c440931-441463 | pilS cassette |
|  |  | NGEG_RS02440 | c441060-441431 | c442710-443207 | pilS cassette |
|  |  | NGEG_RS02315 | c417644-418423 | c417214-417716 | large pilS cassette |
|  |  | NGEG_RS02440 | c441060-441431 | c443208-443593 | pilS cassette |
|  |  | NGEG_RS02495 | c447524-447934 | c447524-447966 | pilS cassette |
|  |  | NGEG_RS02315 | c417644-418423 | c417280-418500 | large pilS cassette |
|  |  | NGEG_RS02480 | c446067-446402 | c446067-446456 | pilS cassette |
|  |  | NGEG_RS02495 | c447524-447934 | c446977-447391 | pilS cassette |
|  |  | NGEG_RS02290 | c414796-415575 | c414378-414795 | large pilS cassette |
|  |  | NGEG_RS02500 | c448491-449171 | c448833-449199 | fimbrial protein |
|  |  | NGEG_RS02290 | c414796-415575 | c414796-415168 | large pilS cassette |
|  |  | NGEG_RS02480 | c446067-446402 | c445028-445533 | pilS cassette |
|  |  | NGEG_RS02440 | c441060-441431 | c441834-442353 | pilS cassette |
|  |  | NGEG_RS02445 | c441995-442354 | c441834-442386 | pilS cassette |
|  |  | NGEG_RS02315 | c417644-418423 | c418070-418500 | large pilS cassette |
|  |  | NGEG_RS08355 | 1540061-1540738 | 1540013-1540738 | opacity-associated protein |
|  |  | NGEG_RS03455 | c625888-626364 | c625831-626412 | Opacity protein opA54 |
|  |  | NGEG_RS01255 | c228123-228806 | c228123-228854 | opacity-associated protein |
|  | NZ_KI391932.1 | NGEG_RS09880 | c166948-167634 | c166948-167682 | opacity-associated protein |
|  | NZ_KI391935.1 | NGEG_RS11295 | 10504-11208 | 10456-11208 | opacity-associated protein |
|  | NZ_KI391936.1 | NGEG_04901 | 10983-11672 | 10935-11672 | opacity-associated protein |
| *N. gonorrhoeae* FA6140 | NZ_EQ972720.1 | NGDG_RS0107315 | 8311-8953 | 8311-8953 | outer membrane preprotein PIIc |
|  | NZ_EQ972724.1 | NGDG_RS0107460 | c1-617 | c1-617 | OpaD |
|  | NZ_EQ972731.1 | NGDG_RS0110410 | c175339-176046 | c175339-176094 | opacity-associated protein |
| *N. gonorrhoeae* i1905 | NZ_JFBA01000012.1 | BK61_RS05845 | 51312-51680 | 51280-51680 | fimbrial protein |
|  | NZ_JFBA01000071.1 | BK61_RS11330 | c641-1072 | c502-1034 | hypothetical protein |
|  |  | BK61_RS11340 | c2593-3134 | c2464-2947 | fimbrial protein |
|  | NZ_JFBA01000083.1 | BK61_RS11570 | 609-1271 | 577-1034 | large pilS cassette |
|  | NZ_JFBA01000091.1 | BK61_RS11655 | c578-931 | c571-931 | hypothetical protein |
|  | NZ_JFBA01000095.1 | BK61_RS11690 | c1-436 | c1-398 | fimbrial protein |
| *N. gonorrhoeae* m07.05 | NZ_JFBB01000008.1 | BK60_RS03450 | 1-656 | 299-795 | large pilS cassette |
|  | NZ_JFBB01000035.1 | BK60_RS08590 | c390-743 | c390-725 | pilin |
|  |  | BK60_RS08585 | c2-298 | c2-349 | pilin |
|  | NZ_JFBB01000046.1 | BK60_11785 | 1-625 | 1-625 | opacity-associated protein |
|  | NZ_JFBB01000086.1 | BK60_RS11055 | c1-436 | c1-366 | hypothetical protein |
|  |  | BK60_RS11065 | c2132-2509 | c2132-2522 | fimbrial protein |
|  | NZ_JFBB01000106.1 | BK60_RS11400 | 212-889 | 164-889 | opacity-associated protein |
|  | NZ_JFBB01000117.1 | BK60_RS11495 | 1-619 | 1-619 | opacity-associated protein |
|  | NZ_JFBB01000120.1 | BK60_RS11515 | c469-822 | c469-822 | fimbrial protein |
| *N. gonorrhoeae* MS11 | CP003909.1 | NGFG_02481 | c2087686-2088063 | c2087591-2088039 | pilin pilS6c1 |
|  |  | NGFG_02405 | 1300336-1300557 | 1300349-1300844 | pilin pilS5 |
|  |  | NGFG_02485 | c2112950-2113369 | c2113465-2113869 | pilin pilS2c1 |
|  |  | NGFG_02484 | c2112950-2113369 | c2112855-2113312 | pilin pilS2c1 |
|  |  | NGFG_02431 | 1553066-1553392 | 1553065-1553487 | pilin pilS7 |
|  |  | NGFG_00014 | c2124213-2125298 | c2124309-2124659 | pilin pilS1c456 |
|  |  | NGFG_01821 | c2109185-2109685 | c2109046-2109685 | pilin pilE |
|  |  | NGFG_00014 | c2124213-2125298 | c2124696-2125043 | pilin pilS1c456 |
|  |  | NGFG_02487 | c2123867-2124205 | c2123906-2124271 | pilin pilS1c3 |
|  |  | NGFG_02253 | c2122673-2123455 | c2122534-2123035 | pilin pilS1c12 |
|  |  | NGFG_02253 | c2122673-2123455 | c2123072-2123479 | pilin pilS1c12 |
|  | NC_022240.1 | NGFG_RS00365 | c69386-70072 | c69540-70276 | opacity-associated protein |
|  |  | NGFG_RS10330 | c1901700-1902377 | c1901868-1902595 | opacity-associated protein |
|  |  | NGFG_RS08610 | 1603480-1604160 | 1603430-1604160 | opacity-associated protein |
|  |  | NGFG_RS11460 | c2107868-2108541 | c2107922-2108645 | Opacity protein opA54 |
|  |  | NGFG_RS08385 | 1553992-1554666 | 1553942-1554666 | opacity-associated protein |
|  |  | NGFG_RS00385 | c74859-75542 | c74913-75646 | opacity-associated protein |
|  |  | NGFG_RS06015 | 1105170-1105856 | 1105120-1105856 | opacity-associated protein |
|  |  | NGFG_RS08090 | c1500420-1501133 | c1500488-1501251 | opacity-associated protein |
|  |  | NGFG_RS05770 | c1063561-1064265 | c1063656-1064414 | opacity-associated protein |
|  |  | NGFG_RS07085 | 1301354-1302055 | 1301304-1302055 | opacity-associated protein |
|  |  | NGFG_RS05305 | 986489-987178 | 986489-987325 | opacity-associated protein |
| *N. gonorrhoeae* n0108 | NZ_JIBZ01000009.1 | DT75_RS05030 | 1-318 | 1-457 | fimbrial protein |
|  |  | DT75_RS05040 | 1632-2009 | 1600-2148 | hypothetical protein |
|  |  | DT75_RS05035 | 823-1296 | 861-1296 | hypothetical protein |
|  | NZ_JIBZ01000023.1 | DT75_RS08420 | c24961-25659 | c24961-25707 | opacity-associated protein |
|  | NZ_JIBZ01000032.1 | DT75_RS09370 | 49-726 | 1-726 | opacity-associated protein |
|  |  | DT75_RS09470 | 20091-20487 | 19837-20378 | pili assembly chaperone |
|  | NZ_JIBZ01000055.1 | DT75_RS11105 | c5163-5852 | c5163-5900 | opacity-associated protein |
|  | NZ_JIBZ01000064.1 | DT75_RS11350 | 1-305 | 1-444 | hypothetical protein |
|  |  | DT75_RS11360 | 1889-2266 | 1472-2405 | hypothetical protein |
|  | NZ_JIBZ01000066.1 | DT75_RS11395 | 1-653 | 1-653 | opacity-associated protein |
|  | NZ_JIBZ01000078.1 | DT75_RS11570 | c631-1332 | c631-1380 | opacity-associated protein |
|  | NZ_JIBZ01000084.1 | DT75_RS11630 | 1-397 | 1-536 | hypothetical protein |
|  | NZ_JIBZ01000098.1 | DT75_RS11715 | 122-401 | 89-401 | fimbrial protein |
| *N. gonorrhoeae* NCCP11945 | NC_011035.1 | NGK_RS09860 | 1816062-1816415 | 1816062-1816415 | pilS cassette |
|  |  | NGK_RS06730 | 1235506-1235880 | 1235506-1236019 | pilin |
|  |  | NGK_RS09745 | 1794061-1794438 | 1794029-1794577 | PilE/pilin (PilE) |
|  |  | NGK_RS09860 | 1816062-1816415 | 1816456-1816895 | pilS cassette |
|  |  | NGK_RS09865 | 1816507-1816803 | 1816436-1816895 | pilin |
| *N. gonorrhoeae* NG05 | NZ_JPOZ01000008.1 | IX30_RS03920 | c63349-63726 | c63210-63756 | hypothetical protein |
|  | NZ_JPOZ01000014.1 | IX30_RS05185 | c1814-2500 | c1814-2548 | opacity-associated protein |
|  | NZ_JPOZ01000032.1 | IX30_RS08500 | c23676-24374 | c23676-24422 | opacity-associated protein |
|  | NZ_JIBZ01000080.1 | DT75_RS11595 | c665-1344 | c665-1344 | opacity-associated protein |
|  | NZ_JPOZ01000087.1 | IX30_RS11275 | 329-1018 | 281-1018 | opacity-associated protein |
|  | NZ_JPOZ01000090.1 | IX30_RS11320 | c665-1378 | c665-1426 | opacity-associated protein |
|  | NZ_JPOZ01000091.1 | IX30_RS11325 | 53-832 | 21-462 | large pilS cassette |
|  |  | IX30_RS11325 | 53-832 | 835-1309 | large pilS cassette |
|  |  | IX30_RS11325 | 53-832 | 463-832 | large pilS cassette |
|  | NZ_JPOZ01000092.1 | IX30_RS11335 | 1-397 | 1-536 | hypothetical protein |
|  |  | IX30_RS11345 | 839-1415 | 791-1415 | opacity-associated protein |
|  | NZ_JPOZ01000095.1 | IX30_RS11375 | c970-1246 | c185-665 | pilin |
|  |  | IX30_RS11375 | c970-1246 | c667-1032 | pilin |
|  | NZ_JPOZ01000101.1 | IX30_RS11440 | c232-525 | c232-590 | hypothetical protein |
|  |  | IX30_RS11445 | c617-965 | c617-965 | hypothetical protein |
|  | NZ_JPOZ01000105.1 | IX30_RS11465 | 43-643 | 43-643 | opacity-associated protein |
|  | NZ_JPOZ01000123.1 | IX30_RS11560 | 1-358 | 17-358 | hypothetical protein |
| *N. gonorrhoeae* PID1 | NZ_EQ972883.1 | NGHG_RS06220 | c93743-94060 | c93097-93438 | pilin |
|  |  | NGHG_RS06110 | c66212-66949 | c66275-66637 | large pilS cassette protein |
|  |  | NGHG_RS06220 | c93743-94060 | c93439-93805 | pilin |
|  |  | NGHG_RS06110 | c66212-66949 | c66639-67003 | large pilS cassette protein |
|  | NZ_EQ972906.1 | NGHG_02137 | 7828-8451 | 7828-8451 | outer membrane protein |
|  | NZ_EQ972920.1 | NGHG_RS15930 | 196-905 | 352-905 | hypothetical protein |
|  |  | NGHG_RS15930 | 196-905 | 223-351 | hypothetical protein |
|  | NZ_EQ972921.1 | NGHG_RS15935 | 183-542 | 151-542 | L-pilin |
|  | NZ_EQ972922.1 | NGHG_RS15940 | 1-466 | 1-331 | fimbrial protein |
|  |  | NGHG_RS15945 | 358-663 | 332-663 | pilS cassette protein |
| *N. gonorrhoeae* PID18 | NZ_EQ972836.1 | NGGG_RS06190 | c65252-65617 | c65696-66045 | L-pilin |
|  |  | NGGG_RS06190 | c65252-65617 | c65252-65643 | L-pilin |
| *N. gonorrhoeae* PID241 | NZ_EQ972931.1 | NGIG_RS0100005 | c13-705 | c13-752 | opacity-associated protein |
|  | NZ_EQ972933.1 | NGIG_RS0100425 | c65430-65720 | c65338-65774 | pilin |
|  |  | NGIG_RS0100425 | c65430-65720 | c65981-66324 | pilin |
|  |  | NGIG_RS0100420 | c63358-63648 | c63219-63658 | hypothetical protein |
|  | NZ_EQ972981.1 | NGIG_RS0111045 | c109-501 | c109-514 | pilS cassette |
| *N. gonorrhoeae* PID332 | NZ_EQ972989.1 | NGJG_RS06870 | c138098-138262 | c138098-138457 | large pilS cassette protein |
|  |  | NGJG_RS06870 | c138098-138262 | c139297-139693 | large pilS cassette protein |
|  |  | NGJG_RS06875 | c138235-138783 | c138458-138837 | large pilS cassette protein |
|  |  | NGJG_RS06990 | c165669-165986 | c165366-165731 | pilin |
|  |  | NGJG_RS06880 | c138876-139661 | c138876-139248 | large pilS cassette |
|  | NZ_EQ972994.1 | NGJG_02236 | c1-673 | c1-673 | outer membrane protein |
|  | NZ_EQ972998.1 | NGJG_RS10035 | 149-694 | 101-694 | opacity-associated protein |
|  | NZ_EQ973009.1 | NGJG_RS13185 | 1-505 | 135-505 | large pilS cassette |
|  | NZ_EQ973017.1 | NGJG_RS14920 | c1-543 | c1-543 | opacity-associated protein |
|  | NZ_EQ973024.1 | NGJG_RS0105775 | 274-636 | 242-638 | hypothetical protein |
| *N. gonorrhoeae* SK92679 | NZ_EQ973035.1 | NGKG_RS114400 | c341338-342024 | c341338-342046 | opacity-associated protein |
|  | NZ_EQ973057.1 | NGKG_02151 | 1-619 | 2-619 | outer membrane protein |
|  | NZ_EQ973070.1 | NGKG_RS01075 | 93-623 | 99-466 | large pilS cassette |
| *N. gonorrhoeae* SK931035 | NZ_EQ973081.1 | NGLG_RS113155 | c1-535 | c1-535 | opacity-associated protein |
|  | NZ_EQ973086.1 | NGLG_RS0102700 | c162683-162973 | c162961-163348 | L-pilin |
|  |  | NGLG_RS08675 | c136335-136667 | c136335-136679 | pilS cassette |
| *N. meningitidis* 053442 | NC_010120.1 | NMCC_RS00125 | c19532-20401 | c20021-20408 | large pilS cassette |
|  |  | NMCC_RS00120 | c19136-19486 | c19132-19504 | pilin family protein |
|  |  | NMO_RS08075 | c1562398-1563792 | 1562773-1563220 | GTP-binding protein |
|  |  | NMCC_RS00125 | c19532-20401 | c19640-20020 | large pilS cassette |
|  |  | NMCC_RS00115 | c18148-18528 | c18010-18541 | fimbrial protein (pilin) |
|  |  | NMCC_RS04530 | c870462-871151 | c870462-871202 | opacity-associated protein |
|  |  | NMCC_RS08945 | 1740550-1741245 | 1740499-1741245 | opacity-associated protein |
|  |  | NMCC_RS07240 | c1397505-1398206 | c1397505-1398257 | opacity-associated protein |
|  |  | NMCC_RS08090 | 1564815-1565480 | 1564764-1565480 | opacity-associated protein |
| *N. meningitidis* 510612 | NZ_CP007524.1 | NMA510612_RS01385 | c254125-254343 | c253985-254440 | fimbrial protein |
|  |  | NMA510612_RS01390 | c254433-254771 | c254443-254795 | fimbrial protein |
|  |  | NMA510612_RS09600 | 1818110-1818769 | 1818059-1818769 | opacity-associated protein |
|  |  | NMA510612_RS08495 | c1611834-1612493 | c1611834-1612544 | hypothetical protein |
|  |  | NMA510612_RS05670 | c1079576-1080265 | c1079576-1080316 | hypothetical protein |
|  |  | NMA510612_RS05670 | c1079576-1080265 | c1079576-1080316 | hypothetical protein |
| *N. meningitidis* 8013 | NC_017501.1 | NMV_RS00100 | c16989-17495 | c16849-17495 | fimbrial protein |
|  |  | NMV_RS00105 | c19501-19920 | c19549-19933 | fimbrial protein |
|  |  | NMV_RS00110 | c20115-20519 | c20163-20532 | fimbrial protein |
|  |  | NMV_RS00115 | c20678-21521 | c20813-21160 | large pilS cassette |
|  |  | NMV_RS07285 | 1434963-1435637 | 1434912-1435637 | opacity protein Opa54 |
|  |  | NMV_RS02435 | c465615-466298 | c465615-466349 | opacity protein Opa54 |
|  |  | NMV_RS03615 | c733165-733860 | c733165-733911 | opacity protein Opa54 |
|  |  | NMV_RS04535 | 915299-915991 | 915248-915991 | opacity protein Opa54 |
| *N. meningitidis* alpha14 | NC_013016.1 | NMO_1984 | 2140287-2140655 | 2140275-2140655 | hypothetical protein |
|  |  | NMO_1986 | 2142220-2142723 | 2142220-2142861 | fimbrial protein |
|  |  | NMO_1980 | 2137569-2138285 | 2137794-2138177 | large PilS cassette |
|  |  | NMO_1985 | 2141148-2141447 | 2141130-2141447 | fimbrial protein |
|  |  | NMO_RS08835 | 1722454-1723113 | 1722403-1723113 | opacity protein Opa54 |
|  |  | NMO_RS08030 | 1552325-1552984 | 1552274-1552984 | opacity protein Opa54 |
|  |  | NMO_RS04485 | c862205-862891 | c862204-862942 | opacity protein Opa54 |
| *N. meningitidis* alpha710 | NC_017505.1 | NMBB_RS00140 | c21973-22497 | c21973-22506 | pilS cassette |
|  |  | NMBB_RS00135 | c21573-21926 | c21573-21926 | pilS cassette |
|  |  | NMBB_RS00125 | c19863-20270 | c19863-20283 | pilS cassette |
|  |  | NMBB_RS00115 | c18508-19023 | c18508-19036 | pilin |
|  |  | NMBB_RS00105 | c17389-17898 | c17250-17898 | fimbrial protein |
|  |  | NMBB_RS00120 | c19037-19441 | c19037-19454 | pilS cassette |
|  |  | NMBB_RS00130 | c21021-21377 | c21021-21390 | pilS cassette |
| *N. meningitidis* FAM18 | NC_008767.1 | NMC_RS00015 | c3341-3628 | c3202-3828 | pilin |
| *N. meningitidis* H44/76 | NC_017516.1 | NMBH4476_RS00125 | c21996-22439 | c21996-22439 | pilus assembly protein PilS |
|  |  | NMBH4476_RS00115 | c20784-21122 | c20490-21146 | fimbrial protein precursor |
|  |  | NMBH4476_RS00100 | c18469-18879 | c18469-18918 | fimbrial protein |
|  |  | NMBH4476_RS00095 | c17190-17693 | c17050-17571 | fimbrial protein |
|  |  | NMBH4476_RS00120 | c21422-21829 | c21422-21842 | fimbrial protein MS11-D3A precursor |
|  |  | NMBH4476_RS00110 | c19870-20244 | c19870-20257 | fimbrial protein |
|  |  | NMBH4476_RS00105 | c19219-19593 | c19125-19637 | pilin family protein |
| *N. meningitidis* LNP21362 | NZ_CP006869.1 | N875_RS06755 | c1286013-1286456 | c1286091-1286456 | pilus assembly protein PilS |
|  |  | N875_RS06735 | c1283657-1284067 | c1283657-1284080 | fimbrial protein |
|  |  | N875_RS06730 | c1282378-1282881 | c1282238-1282881 | fimbrial protein |
|  |  | N875_RS06755 | c1286013-1286456 | c1285439-1285859 | pilus assembly protein PilS |
|  |  | N875_RS06740 | c1284407-1284781 | c1284407-1284794 | pilin family protein |
| *N. meningitidis* M01240149 | NC_017514.1 | NMBM01240149_RS00110 | c19842-20189 | c19842-20240 | hypothetical protein |
|  |  | NMBM01240149_RS00105 | c19181-19561 | c19181-19574 | pilS cassette |
| *N. meningitidis* M01240355 | NC_017517.1 | NMBM01240355_RS00095 | c16623-17135 | c16485-17135 | fimbrial protein |
|  |  | NMBM01240355_RS00100 | c18229-18609 | c18229-18609 | pilS cassette |
| *N. meningitidis* M04240196 | NC_017515.1 | NMBM04240196_RS00100 | c17737-18099 | c17584-18129 | pilin |
| *N. meningitidis* M10208 | NZ_CP009422.1 | LD07_RS04520 | c886184-886471 | c886045-886631 | pilin |
| *N. meningitidis* M7124 | NZ_CP009419.1 | LA24_RS00860 | 179672-179959 | 179512-180098 | pilin |
| *N. meningitidis* MC58 | NC_003112.2 | NMB0024 | c21891-22487 | c21891-22487 | fimbrial protein |
|  |  | NMB0025 | c22825-23502 | c23183-23502 | fimbrial protein |
|  |  | NMB0019 | c18517-18927 | c18517-18940 | fimbrial protein |
|  |  | NMB0023 | c21470-21877 | c21470-21879 | fimbrial protein |
|  |  | NMB0018 | c17229-17741 | c17089-17741 | pilE |
|  |  | NMB0025 | c22825-23502 | c22825-23181 | fimbrial protein |
|  |  | NMB0021 | c19918-20292 | c19918-20294 | fimbrial protein |
|  |  | NMB0020 | c19267-19641 | c19267-19654 | fimbrial protein |
|  |  | NMB0019 | c18517-18927 | c18517-18940 | fimbrial protein |
| *N. meningitidis* NM3682 | NZ_CP009420.1 | LA58_RS09630 | 1865885-1866172 | 1865725-1866311 | pilin |
| *N. meningitidis* NM3683 | NZ_CP009421.1 | LC14_RS11175 | c2150103-2150390 | c2149964-2150550 | pilin |
| *N. meningitidis* NZ-05/33 | NC_017518.1 | NMBNZ0533_RS00150 | c23276-23770 | c23076-23779 | pilS cassette |
|  |  | NMBNZ0533_RS00155 | c24009-24368 | c24009-24392 | pilin family protein |
|  |  | NMBNZ0533_RS00130 | c21388-21798 | c21388-21811 | fimbrial protein |
|  |  | NMBNZ0533_RS00110 | c18689-19096 | c18689-19109 | fimbrial protein |
|  |  | NMBNZ0533_RS00105 | c17412-17912 | c17272-17912 | fimbrial protein |
|  |  | NMBNZ0533_RS00115 | c19477-19800 | c19477-19813 | fimbrial protein |
|  |  | NMBNZ0533_RS00145 | c22975-23322 | c22975-23770 | fimbrial protein |
|  |  | NMBNZ0533_RS00135 | c22153-22527 | c22153-22540 | fimbrial protein |
|  |  | NMBNZ0533_RS00120 | c20154-20534 | c20154-20541 | fimbrial protein |
|  |  | NMBNZ0533_RS00140 | c22606-22929 | c22606-22929 | pilS cassette |
|  |  | NMBNZ0533_RS00125 | c20773-21192 | c20773-21205 | fimbrial protein |
| *N. meningitidis* WUE2594 | NC_017512.1 | NMAA_RS00105 | c17073-17411 | c17073-17435 | fimbrial protein |
|  |  | NMAA_RS07235 | c1376007-1376666 | c1376007-1376717 | hypothetical protein |
| *N. meningitidis* Z2491 | NC_003116.1 | NMA_RS01425 | c256171-256491 | c256171-256491 | pilin family protein |
|  |  | NMA_RS01420 | c255766-256125 | c255766-256125 | pilin family protein |
|  |  | NMA_RS01390 | c250476-250988 | c250336-250988 | fimbrial protein |
|  |  | NMA_RS01400 | c252682-253101 | c252682-253108 | fimbrial protein |
|  |  | NMA_RS01415 | c255227-255643 | c255227-255653 | pilin family protein |
|  |  | NMA_RS01405 | c253273-253641 | c253273-253644 | pilin family protein |
|  |  | NMA_RS01395 | c252156-252497 | c252156-252549 | hypothetical protein |
|  |  | NMA_RS08415 | c1599569-1600246 | c1599569-1600297 | opacity-associated protein |
|  |  | NMA_RS09545 | 1815357-1816049 | 1815306-1816049 | opacity-associated protein |
