# Supplementary material for: Positive Selection Pressure Drives Variation on the Surface-Exposed Variable Proteins of the Pathogenic Neisseria
Source: PLoS One. 2016 Aug 17;11(8):e0161348. doi: 10.1371/journal.pone.0161348 (PMC5020929; doi:10.1371/journal.pone.0161348)
Supplement: S2 Table — (DOCX) [file pone.0161348.s007.docx]

**S2 Supplemental Table. Test of substitution saturation of the *opa* and *pil* nucleotide alignments.**

| Gene | *N*_OTU_ | I_SS_ | I_SS.C_ | T | DF | P |
| --- | --- | --- | --- | --- | --- | --- |
| *opa* | 4 | 0.273 | 0.760 | 15.041 | 242 | < 0.0001 |
| *pil* | 4 | 0.133 | 0.770 | 23.803 | 191 | < 0.0001 |
