# Supplementary material for: Positive Selection Pressure Drives Variation on the Surface-Exposed Variable Proteins of the Pathogenic Neisseria
Source: PLoS One. 2016 Aug 17;11(8):e0161348. doi: 10.1371/journal.pone.0161348 (PMC5020929; doi:10.1371/journal.pone.0161348)
Supplement: S3 Table — (DOCX) [file pone.0161348.s008.docx]

**S3 Supplemental Table.**

| Species | Strain | Possible Pairwise Comparisons | Polymorphic Sites | Statistically Significant Pairwise Comparisons | D′ |
| --- | --- | --- | --- | --- | --- |
| *N. meningitidis* |  |  |  |  |  |
|  | 053442  (*n* = 4) | 7021 | 119 | 0 |  |
|  | 510612  (*n* = 3) | 6786 | 117 | 0 |  |
|  | alpha14  (*n* = 3) | 2346 | 69 | 0 |  |
|  | Z2491  (*n* = 2) | 1711 | 59 | 0 |  |
| *N. gonorrhoeae* |  |  |  |  |  |
|  | 8013  (*n* = 4) | 3240 | 81 | 0 |  |
|  | DGI18  (*n* = 2) | 8385 | 130 | 0 |  |
|  | e03.04  (*n* = 4) | 5778 | 108 | 0 |  |
|  | F62  (*n* = 6) | 6786 | 117 | 0 |  |
|  | FA1090  (*n* = 11) | 9730 | 140 | 555 | 0.06 |
|  | FA19  (*n* = 8) | 10153 | 143 | 282 | 0.03 |
|  | FA6140  (*n* = 3) | 10731 | 147 | 0 |  |
|  | m07.05  (*n* = 3) | 8911 | 134 | 0 |  |
|  | MS11  (*n* = 11) | 8385 | 130 | 493 | 0.06 |
|  | n01.08  (*n* = 6) | 8778 | 133 | 0 |  |
|  | NG05  (*n* = 6) | 13041 | 162 | 0 |  |
|  | PID332  (*n* = 3) | 9591 | 139 | 0 |  |
|  | SK-93-1035  (*n* = 2) | 1953 | 63 | 0 |  |
