# Supplementary material for: Positive Selection Pressure Drives Variation on the Surface-Exposed Variable Proteins of the Pathogenic Neisseria
Source: PLoS One. 2016 Aug 17;11(8):e0161348. doi: 10.1371/journal.pone.0161348 (PMC5020929; doi:10.1371/journal.pone.0161348)
Supplement: S4 Table — (DOCX) [file pone.0161348.s009.docx]

**S4 Supplemental Table.**

| Species | Strain | Possible Pairwise Comparisons | Polymorphic Sites | Statistically Significant Pairwise Comparisons | D′ |
| --- | --- | --- | --- | --- | --- |
| *N. meningitidis* |  |  |  |  |  |
|  | 053442  (*n* = 5) | 2850 | 76 | 0 |  |
|  | 3502  (*n* = 6) | 2926 | 77 | 0 |  |
|  | 510612  (*n* = 2) | 1081 | 47 | 0 |  |
|  | alpha14  (*n* = 4) | 4851 | 99 | 0 |  |
|  | alpha710  (*n* = 7) | 2701 | 74 | 78 | 0.03 |
|  | H44/76  (*n* = 7) | 3828 | 88 | 121 | 0.03 |
|  | LNP21362  (*n* = 5) | 3486 | 84 | 0 |  |
|  | M01-240149  (*n* = 2) | 1540 | 56 | 0 |  |
|  | M01-240355  (*n* = 2) | 666 | 37 | 0 |  |
|  | MC58  (*n* = 8) | 4656 | 97 | 177 | 0.04 |
|  | NZ-05/33  (*n* = 11) | 1711 | 59 | 187 | 0.11 |
|  | Z2491  (*n* = 7) | 3081 | 79 | 147 | 0.05 |
| *N. gonorrhoeae* |  |  |  |  |  |
|  | 8013  (*n* = 4) | 1326 | 52 | 0 |  |
|  | DGI2  (*n* = 9) | 3916 | 89 | 100 | 0.03 |
|  | DGI18  (*n* = 5) | 2278 | 68 | 0 |  |
|  | e03.04  (*n* = 6) | 4560 | 96 | 0 |  |
|  | F62  (*n* = 11) | 3655 | 86 | 55 | 0.02 |
|  | FA1090  (*n* = 19) | 3003 | 78 | 103 | 0.03 |
|  | FA19  (*n* = 21) | 2628 | 73 | 144 | 0.05 |
|  | i19.05  (*n* = 6) | 2850 | 76 | 0 |  |
|  | m07.05  (*n* = 6) | 2850 | 76 | 0 |  |
|  | MS11  (*n* = 11) | 3741 | 87 | 59 | 0.02 |
|  | n01.08  (*n* = 8) | 2415 | 70 | 30 | 0.01 |
|  | NCCP11945  (*n* = 5) | 3828 | 88 | 0 |  |
|  | NG05  (*n* = 10) | 3570 | 85 | 76 | 0.02 |
|  | PID1  (*n* = 9) | 2278 | 68 | 35 | 0.02 |
|  | PID18  (*n* = 2) | 990 | 45 | 0 |  |
|  | PID24-1  (*n* = 4) | 1540 | 56 | 0 |  |
|  | PID332  (*n* = 7) | 3655 | 86 | 33 | 0.01 |
|  | SK-93-1035  (*n* = 2) | 1378 | 53 | 0 |  |
