# Supplementary material for: Positive Selection Pressure Drives Variation on the Surface-Exposed Variable Proteins of the Pathogenic Neisseria
Source: PLoS One. 2016 Aug 17;11(8):e0161348. doi: 10.1371/journal.pone.0161348 (PMC5020929; doi:10.1371/journal.pone.0161348)
Supplement: S5 Table — Genes are shown along with the strain of Mc or Gc and the number of sequences that were analyzed. The value of γ and the p value for that estimate are also shown. (DOCX) [file pone.0161348.s010.docx]

**S5 Supplemental Table.**

| Gene | Intrastrain | γ (95% confidence interval) | Significance (p-value) | Outcome |
| --- | --- | --- | --- | --- |
| *pil*  *n* = 219 |  | 3.44 + 3.11 | 3.78 x 10^-5^ | Indicates selection |
|  | *N. meningitidis* 8013  *n* = 4 | 2.77+ 8.30 | 2.71 x 10^-1^ | Does not indicate selection |
|  | *N. meningitidis* 053442  *n* = 5 | 2.77+ 6.65 | 1.62 x 10^-1^ | Does not indicate selection |
|  | *N. meningitidis* 510612  *n* = 2 | All polymorphisms are singletons |  | Does not indicate selection |
|  | *N. meningitidis* alpha14  *n* = 4 | 2.77+ 7.22 | 2.05 x 10^-1^ | Does not indicate selection |
|  | *N. meningitidis* alpha710  *n* = 7 | 2.72+ 4.49 | 4.73 x 10^-2^ | Indicates selection |
|  | *N. meningitidis* H44/76  *n* = 7 | 2.75+ 4.82 | 5.65 x 10^-2^ | Does not indicate selection |
|  | *N. meningitidis* LNP21362  *n* = 5 | 2.77+ 7.11 | 1.90 x 10^-1^ | Does not indicate selection |
|  | *N. meningitidis* M0120149  *n* = 2 | All polymorphisms are singletons |  | Does not indicate selection |
|  | *N. meningitidis* M0124355  *n* = 2 | All polymorphisms are singletons |  | Does not indicate selection |
|  | *N. meningitidis* MC58  *n* = 8 | 2.79+ 3.76 | 1.45 x 10^-2^ | Indicates selection |
|  | *N. meningitidis* NZ-05/33  *n* = 11 | 2.94+ 4.29 | 1.90 x 10^-2^ | Indicates selection |
|  | *N. meningitidis* Z2491  *n* = 7 | 2.73+ 3.65 | 1.57 x 10^-2^ | Indicates selection |
|  | *N. gonorrhoeae*  3502  *n* = 6 | 2.76 + 4.54 | 4.64 x 10^-2^ | Indicates selection |
|  | *N. gonorrhoeae*  DG12  *n* = 9 | 2.78 + 3.11 | 3.77 x 10^-3^ | Indicates selection |
|  | *N. gonorrhoeae*  DG18  *n* = 5 | 2.77 + 6.37 | 1.48 x 10^-1^ | Does not indicate selection |
|  | *N. gonorrhoeae*  e0304  *n* = 6 | 2.72 + 4.30 | 3.91 x 10^-2^ | Indicates selection |
|  | *N. gonorrhoeae*  F62  *n* = 11 | 2.63 + 2.79 | 2.71 x 10^-3^ | Indicates selection |
|  | *N. gonorrhoeae*  FA19  *n* = 21 | 2.82 + 2.80 | 8.67 x 10^-4^ | Indicates selection |
|  | *N. gonorrhoeae*  FA1090  *n* = 19 | 2.71 + 2.66 | 2.04 x 10^-3^ | Indicates selection |
|  | *N. gonorrhoeae*  i1905  *n* = 6 | 2.71 + 4.68 | 5.86 x 10^-2^ | Does not indicate selection |
|  | *N. gonorrhoeae*  m07.05  *n* = 6 | 2.70 + 3.86 | 2.41 x 10^-2^ | Indicates selection |
|  | *N. gonorrhoeae*  MS11  *n* = 11 | 2.83 + 3.08 | 2.54 x 10^-3^ | Indicates selection |
|  | *N. gonorrhoeae*  n01.08  *n* = 8 | 2.72 + 3.93 | 2.51 x 10^-2^ | Indicates selection |
|  | *N. gonorrhoeae*  NCCP11945  *n* = 5 | 2.77 + 4.80 | 5.84 x 10^-2^ | Does not indicate selection |
|  | *N. gonorrhoeae*  NG05  *n* = 10 | 2.64 + 2.88 | 3.59 x 10^-3^ | Indicates selection |
|  | *N. gonorrhoeae*  PID1  *n* = 9 | 2.64 + 3.28 | 1.05 x 10^-2^ | Indicates selection |
|  | *N. gonorrhoeae*  PID18  *n* = 2 | All polymorphisms are singleton |  | Does not indicate selection |
|  | *N. gonorrhoeae*  PID241  *n* = 4 | 2.77 + 8.30 | 2.71 x 10^-1^ | Does not indicate selection |
|  | *N. gonorrhoeae*  PID332  *n* = 7 | 2.78 + 4.14 | 2.67 x 10^-2^ | Indicates selection |
|  | *N. gonorrhoeae*  SK-93-1035  *n* = 2 | All polymorphisms are singletons |  | Does not indicate selection |
|  | *N. gonorrhoeae*  3502  *n* = 6 | 2.76 + 4.54 | 4.64 x 10^-2^ | Indicates selection |
| *opa*  *n* = 86 |  | 3.44 + 3.11 | 3.78 x 10^-5^ | Indicates selection |
|  | *N. meningitidis* 8013  *n* = 4 | 2.77 + 5.27 | 8.67 x 10^-2^ | Does not indicate selection |
|  | *N. meningitidis* 053442  *n* = 4 | 2.77 + 18.73 | 5.58 x 10^-1^ | Does not indicate selection |
|  | *N. meningitidis* 516012  *n* = 3 | All polymorphisms are singletons |  | Does not indicate selection |
|  | *N. meningitidis* alpha14  *n* = 3 | All polymorphisms are singletons |  | Does not indicate selection |
|  | *N. meningitidis* Z2491  *n* = 2 | All polymorphisms are singletons |  | Does not indicate selection |
|  | *N. gonorrhoeae*  DGI18  *n* = 2 | All polymorphisms are singletons |  | Does not indicate selection |
|  | *N. gonorrhoeae*  e0304  *n* = 4 | 2.77 + 5.55 | 1.00 x 10^-1^ | Does not indicate selection |
|  | *N. gonorrhoeae*  F62  *n* = 6 | 2.71 + 3.53 | 1.29 x 10^-2^ | Indicates selection |
|  | *N. gonorrhoeae*  FA19  *n* = 8 | 2.87 + 3.75 | 9.07 x 10^-3^ | Indicates selection |
|  | *N. gonorrhoeae*  FA1090  *n* = 11 | 2.86 + 2.48 | 1.30 x 10^-4^ | Indicates selection |
|  | *N. gonorrhoeae*  FA6140  *n* = 3 | All polymorphisms are singletons |  | Does not indicate selection |
|  | *N. gonorrhoeae*  m07.05  *n* = 3 | All polymorphisms are singletons |  | Does not indicate selection |
|  | *N. gonorrhoeae*  MS11  *n* = 11 | 2.81 + 3.42 | 5.35 x 10^-3^ | Indicates selection |
|  | *N. gonorrhoeae*  n01.08  *n* = 8 | 2.72 + 3.93 | 2.51 x 10^-2^ | Indicates selection |
|  | *N. gonorrhoeae*  NCCP11945  *n* = 5 | 2.77 + 4.80 | 5.84 x 10^-2^ | Does not indicate selection |
|  | *N. gonorrhoeae*  NG05  *n* = 10 | 2.64 + 2.88 | 3.59 x 10^-3^ | Indicates selection |
|  | *N. gonorrhoeae*  PID1  *n* = 9 | 2.64 + 3.28 | 1.05 x 10^-2^ | Indicates selection |
|  | *N. gonorrhoeae*  PID18  *n* = 2 | All polymorphisms are singletons |  | Does not indicate selection |
|  | *N. gonorrhoeae*  PID241  *n* = 4 | 2.77 + 8.30 | 2.71 x 10^-1^ | Does not indicate selection |
|  | *N. gonorrhoeae*  PID332  *n* = 7 | 2.78 + 4.14 | 2.67 x 10^-2^ | Does not indicate selection |
|  | *N. gonorrhoeae*  SK-93-1035  *n* = 2 | All polymorphisms are singletons |  | Does not indicate selection |
