# Supplementary material for: Positive Selection Pressure Drives Variation on the Surface-Exposed Variable Proteins of the Pathogenic Neisseria
Source: PLoS One. 2016 Aug 17;11(8):e0161348. doi: 10.1371/journal.pone.0161348 (PMC5020929; doi:10.1371/journal.pone.0161348)
Supplement: S6 Table — (DOCX) [file pone.0161348.s011.docx]

**S6 Supplemental Table.**

|  |  | Amino acid properties | | | | |  |
| --- | --- | --- | --- | --- | --- | --- | --- |
| Average solvent-exposed surface area | Location | Positive | Negative | Polar | Hydrophobic | Special cases | Amino acids predicted to be under positive selection |
| <20%* |  |  |  |  |  |  | 9.52% |
|  | Entire protein | 4.38% | 4.73% | 5.48% | 7.33% | 3.57% |  |
|  | Membrane spanning | 1.53% | 2.11% | 1.33% | 3.76% | 2.05% |  |
|  | HV1 | 0.53% | 0.31% | 0.12% | 0.32% | 0.00% |  |
|  | HV2 | 0.21% | 0.45% | 1.02% | 0.45% | 0.41% |  |
|  | Semivariable | 0.05% | 0.31% | 0.45% | 0.36% | 0.05% |  |
| 20-50% |  |  |  |  |  |  | 38.10% |
|  | Entire protein | 6.86% | 2.50% | 7.48% | 10.94% | 4.74% |  |
|  | Membrane spanning | 1.77% | 0.15% | 0.44% | 4.25% | 1.51% |  |
|  | HV1 | 0.69% | 0.46% | 1.04% | 0.28% | 0.27% |  |
|  | HV2 | 1.21% | 0.21% | 2.16% | 1.40% | 0.54% |  |
|  | Semivariable | 0.42% | 0.06% | 0.70% | 0.30% | 0.58% |  |
| >50%^ |  |  |  |  |  |  | 52.38% |
|  | Entire protein | 7.99% | 1.97% | 10.40% | 17.34% | 4.28% |  |
|  | Membrane spanning | 0.45% | 0.00% | 0.65% | 9.52% | 0.47% |  |
|  | HV1 | 1.69% | 0.53% | 3.22% | 1.97% | 0.28% |  |
|  | HV2 | 2.82% | 0.94% | 3.95% | 3.18% | 1.54% |  |
|  | Semivariable | 0.36% | 0.07% | 0.22% | 0.29% | 0.20% |  |

*Amino acid is predicted to be buried within the final protein structure.

^Amino acid is predicted to be solvent exposed.
