# Supplementary material for: Positive Selection Pressure Drives Variation on the Surface-Exposed Variable Proteins of the Pathogenic Neisseria
Source: PLoS One. 2016 Aug 17;11(8):e0161348. doi: 10.1371/journal.pone.0161348 (PMC5020929; doi:10.1371/journal.pone.0161348)
Supplement: S7 Table — (DOCX) [file pone.0161348.s012.docx]

**S7 Supplemental Table.**

|  |  | Amino acid properties | | | | |  |
| --- | --- | --- | --- | --- | --- | --- | --- |
| Average solvent-exposed surface area | Location | Positive | Negative | Polar | Hydrophobic | Special cases | Amino acids predicted to be under positive selection |
| <20%* |  |  |  |  |  |  | 10.26% |
|  | Entire protein | 3.47% | 0.17% | 5.49% | 22.72% | 7.99% |  |
|  | mc6 | 0.01% | 0.00% | 1.43% | 2.24% | 1.13% |  |
|  | mc5 | 0.00% | 0.00% | 0.00% | 4.29% | 0.00% |  |
|  | mc4 | 0.09% | 0.15% | 1.47% | 2.57% | 0.00% |  |
|  | mc3 | 0.85% | 0.00% | 0.00% | 0.01% | 0.00% |  |
|  | mc2 | 0.84% | 0.00% | 0.03% | 1.65% | 0.00% |  |
|  | mc1 | 0.00% | 0.00% | 0.00% | 0.00% | 0.00% |  |
| 20-50% |  |  |  |  |  |  | 35.90% |
|  | Entire protein | 9.66% | 7.28% | 11.77% | 6.10% | 4.58% |  |
|  | mc6 | 0.73% | 0.65% | 1.37% | 0.96% | 0.99% |  |
|  | mc5 | 1.51% | 0.67% | 1.56% | 0.31% | 1.09% |  |
|  | mc4 | 1.48% | 0.86% | 0.81% | 0.50% | 0.63% |  |
|  | mc3 | 0.85% | 0.68% | 0.83% | 0.20% | 0.01% |  |
|  | mc2 | 0.97% | 3.14% | 2.00% | 1.84% | 0.63% |  |
|  | mc1 | 0.74% | 1.05% | 2.56% | 1.38% | 0.59% |  |
| >50%^ |  |  |  |  |  |  | 53.85% |
|  | Entire protein | 3.36% | 3.93% | 8.10% | 3.68% | 1.70% |  |
|  | mc6 | 0.44% | 1.18% | 0.84% | 0.42% | 0.46% |  |
|  | mc5 | 1.57% | 0.94% | 1.36% | 0.51% | 0.13% |  |
|  | mc4 | 0.00% | 0.18% | 1.47% | 0.00% | 0.06% |  |
|  | mc3 | 0.00% | 0.00% | 0.00% | 0.00% | 0.00% |  |
|  | mc2 | 1.08% | 1.33% | 2.53% | 1.93% | 0.62% |  |
|  | mc1 | 0.27% | 0.29% | 0.20% | 0.81% | 0.42% |  |

*Amino acid is predicted to be buried within the final protein structure.

^Amino acid is predicted to be solvent exposed.
